# Gene regulatory network analysis predicts cooperating transcription factor regulons required for FLT3-ITD+ AML growth

**DOI:** 10.1016/j.celrep.2023.113568

**Published:** 2023-12-15

**Authors:** Daniel J.L. Coleman, Peter Keane, Rosario Luque-Martin, Paulynn S. Chin, Helen Blair, Luke Ames, Sophie G. Kellaway, James Griffin, Elizabeth Holmes, Sandeep Potluri, Salam A. Assi, John Bushweller, Olaf Heidenreich, Peter N. Cockerill, Constanze Bonifer

**Affiliations:** 1 Institute of Cancer and Genomic Sciences, College of Medicine and Dentistry, University of Birmingham, Edgbaston, Birmingham B15 2TT, UK; 2 School of Biosciences, University of Birmingham, Birmingham B15 2TT, U.K.; 3 Wolfson Childhood Cancer Research Centre, Translational and Clinical Research Institute, Newcastle University, Herschel Building, Level 6, Brewery Lane, Newcastle upon Tyne NE1 7RU, UK; 4 Prinses Máxima Centrum for Pediatric Oncology, Postbus 113, 3720 AC Bilthoven, Heidelberglaan 25, 3584CS Utrecht, the Netherlands; 5 University of Virginia, 1340 Jefferson Park Avenue, Charlottesville, VA 22908, USA; 6 These authors contributed equally; 7 These authors contributed equally; 8 Lead contact

## Abstract

Acute myeloid leukemia (AML) is a heterogeneous disease caused by different mutations. Previously, we showed that each mutational subtype develops its specific gene regulatory network (GRN) with transcription factors interacting within multiple gene modules, many of which are transcription factor genes themselves. Here, we hypothesize that highly connected nodes within such networks comprise crucial regulators of AML maintenance. We test this hypothesis using FLT3-ITD-mutated AML as a model and conduct an shRNA drop-out screen informed by this analysis. We show that AML-specific GRNs predict crucial regulatory modules required for AML growth. Furthermore, our work shows that all modules are highly connected and regulate each other. The careful multi-omic analysis of the role of one (RUNX1) module by shRNA and chemical inhibition shows that this transcription factor and its target genes stabilize the GRN of FLT3-ITD+ AML and that its removal leads to GRN collapse and cell death.

## In brief

Coleman et al. identify a robust gene regulatory network for patients with FLT3-ITD AML, highlighting a crucial role of several different transcription factors including RUNX1 in FLT3-ITD pathology and identifying drug-responsive AML-subtype-specific and overlapping regulatory modules. Their data show identifying AML-subtype-specific GRNs predicts genes required for AML maintenance.

## Graphical abstract

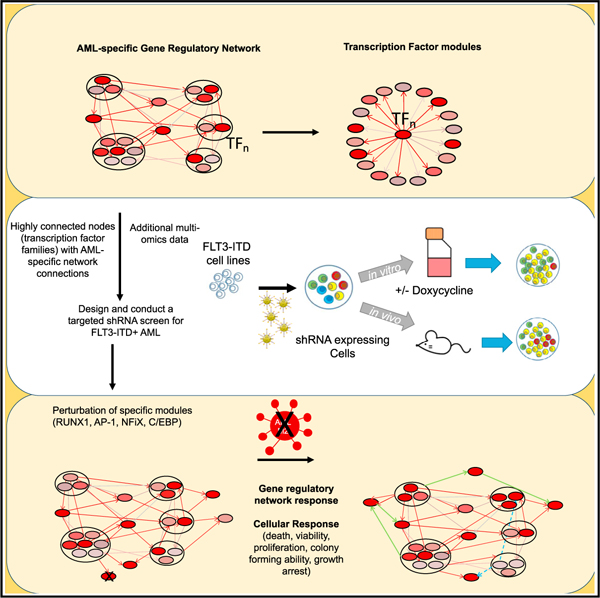

## INTRODUCTION

Cancer occurs when mutations in signaling genes, transcription factors (TFs), and epigenetic regulators cause a block in the normal program of differentiation and an increase in proliferation. Acute myeloid leukemia (AML) is no exception, and due to the clonal nature of the disease, different driver mutations of the cancer cause distinct patterns of gene regulation in AML cells.^1,2^ Due to the differing effects of these mutations, there is a drive to identify druggable targets for each distinct AML subtype to tailor treatment to the individual and to reduce the need for intensive chemotherapy, which is often not tolerated by elderly patients.

AML with an internal tandem duplication of the FLT3 receptor (FLT3-ITD+ AML), which converts a ligand-responsive receptor into a constitutively active molecule, is a highly aggressive AML subtype that is frequently refractory to first line therapy.^3^ The FLT3-ITD mutation occurs on the background of other mutations, mostly in epigenetic regulators such as DNMT3 or TET2, or as a result of clonal evolution.^4,5^ For reasons that are as yet unknown, this mutation often occurs together with a mutation in nucleophosmin (NPM1). Less than 60% of patients with FLT3-ITD+ AML who are over 60 years old reach complete remission and often relapse within a year with an overall relapse rate of 77%.^6^ For this reason, therapy efforts have focused on the development of improved FLT3-specific inhibitors, resulting in the approval of gilteritinib for use in a clinical setting. However, relapse still frequently occurs after treatment with these inhibitors, often due to activating mutations in other signaling genes.^7^ The identification of other druggable targets in FLT3-ITD+ AML is therefore necessary to improve the outcome of patients. To this end, various efforts have been conducted to employ genome-wide RNAi or CRISPR-Cas9 screens that highlighted numerous targets.^8,9^ However, a caveat of such screens is often that they come up with a large number of targets, many of which are also important for normal cells, and they often require follow-up experiments to identify therapeutic windows for their inhibition. To home in on the true AML-sub-type-specific targets, it is therefore necessary to elucidate the fine details of the molecular mechanisms driving AML growth and maintenance of each subtype.

Differential gene expression and thus cellular identity are controlled by the regulatory interactions between TFs and their target genes, which form extensive interacting gene regulatory networks (GRNs). We have previously shown that AML blast cells with FLT3-ITD and FLT3-ITD/NPM1 mutations display a specific chromatin signature distinct from healthy CD34^+^ cells.^1^ To construct GRNs, we then integrated transcriptomic (RNA-seq), HiC, and digital footprinting data based on high-resolution DNaseI-seq experiments.^2^ The comparison between the GRNs of normal and malignant cells identified TF families showing FLT3-ITD+ AML-subtype-specific interactions with their targets, which could be attributed to aberrant expression of genes promoting AML survival. For several of these TFs, such as the AP-1 TF family, we could indeed show that they were required for the maintenance of AML but not normal cells. For example, inhibiting AP-1 activity completely blocked tumorigenesis in two different patient-derived xenograft (PDX) mouse models.^2^ However, the molecular details of how specific TFs contribute to the maintenance of specific AML subtypes and their interaction with genes encoding other TFs is largely unclear.

To answer these questions, we generated a refined GRN for FLT3-ITD and FLT3-ITD/NPM1 AML. Based on this analysis, we designed a targeted shRNA screen to interrogate the contribution of specific TF network nodes and their downstream targets to AML establishment and maintenance. These analyses highlight a crucial role of several different TFs including RUNX1 in FLT3-ITD pathology and identify drug-responsive AML-sub-type-specific and overlapping regulatory modules. Taken together, our data show that identifying AML-subtype-specific GRNs is predictive for genes required for AML maintenance.

## RESULTS

### Constructing a refined FLT3-ITD+ AML GRN

**Figure 1.**
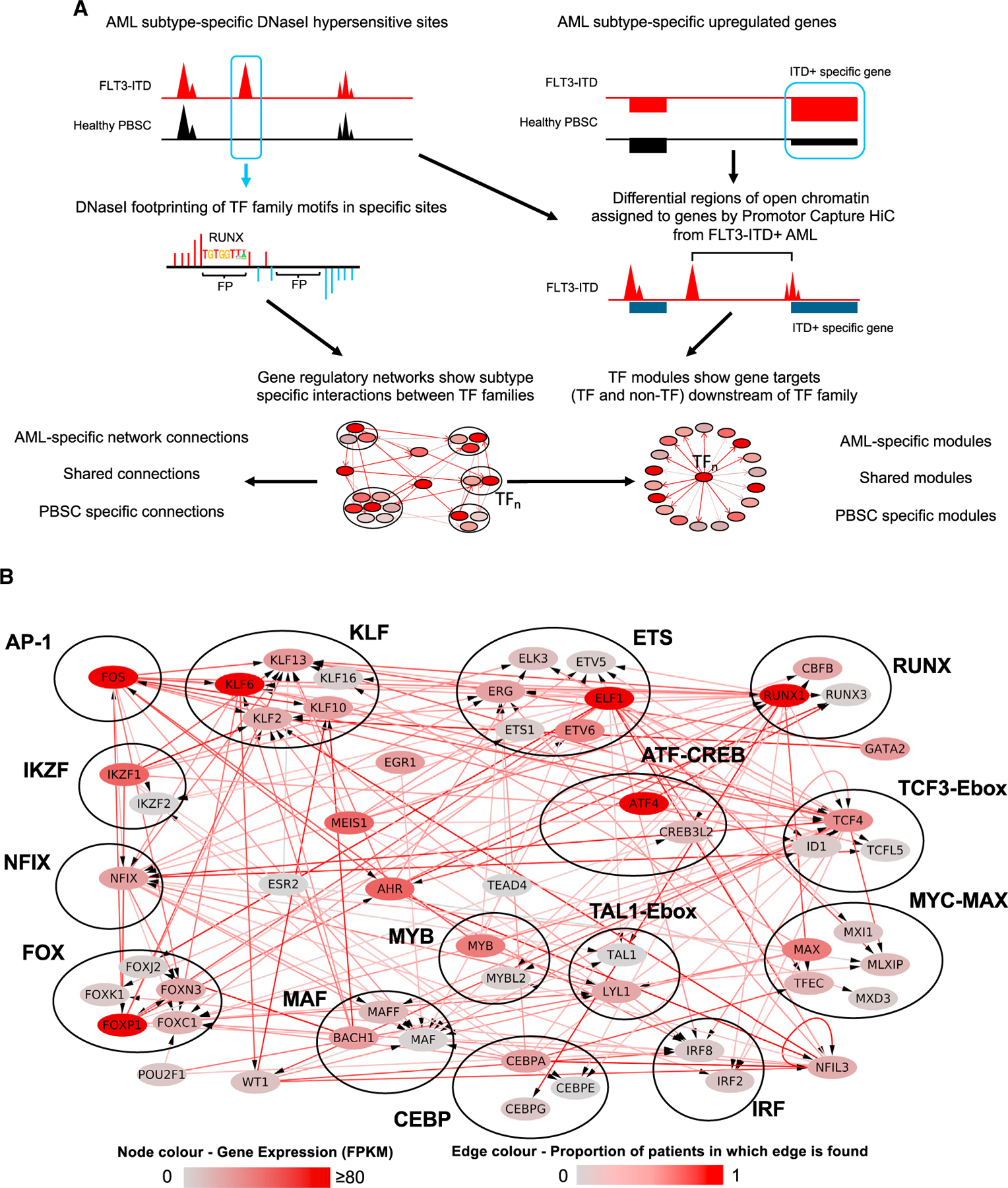
A refined gene regulatory network for FLT3-ITD and FLT3-ITD/NPM1 AML (A) Scheme of transcription factor (TF) network and regulatory module generation. (B) FLT3-ITD/FLT3-ITD NPM1 TF network generated by integrating data from DHSs specific for FLT3-ITD/FLT3-ITD NPM1 AML identified in DHSs from nine patients compared to healthy PBSCs. TF families binding to the same motif are encircled. The color code for nodes and edges is explained at the bottom of the figure. Edges indicate the presence of a TF motif in a region of chromatin open in FLT3-ITD+ AML and assigned to the target gene by promoter capture HiC or the nearest gene (<200 kb). Edge color represents the proportion of patients in which that edge could be found. A high value indicates that an edge is found in multiple patients with AML, suggesting a high level of support for that edge. Only genes with connections from TF families are shown; if there are no incoming connections from other TF families, then the highest expressed gene in the TF family in FLT3-ITD+ AML is shown (see also Figure S1).

Cell-type-specific gene expression is largely encoded in the distal enhancer elements physically interacting with their cognate promoters.^10^ Our previously constructed GRNs for AML with FLT3-ITD and FLT3-ITD/NPM1 genotypes highlighted interactions between upregulated TF families and distal elements demonstrating that each AML-subtype displayed a specific gene regulatory phenotype distinct from that of normal cells and of each other. However, to demonstrate the full regulatory relevance of the GRN and its downstream targets for one specific AML subtype, we need to examine the complete GRN. Here we developed a new computational script that is open access and uses our published data to construct a full GRN that contains the full set of regulatory interactions between all *cis* regulatory elements of all genes including promoters of all genes, as depicted schematically in Figure 1A (see STAR Methods). The result of such an analysis can then be filtered to visualize individual motifs and sub-networks. Briefly, open chromatin regions specific to AML (>3-fold change [FC] compared to normal cells) were determined by analyzing high-read-depth DNaseI-seq data. Occupied TF binding motifs as determined by digital footprinting within such sites were then assigned to a TF family.^2,11^ Such regions were assigned to promotors using previously determined promoter-capture HiC (CHiC) interactions from a FLT3-ITD+ AML sample or the nearest gene (both within 200 kb of the promoter).^2^ RNA-seq was used to identify genes specifically upregulated in the FLT3-ITD+ AML subtype compared to healthy CD34^+^ cells (>2 FC, p < 0.1). These data were plotted as a network showing specific interactions between TF families and their target genes in FLT3-ITD+ AML. Many of these targets are TF-encoding genes themselves, and it is these targets that are shown here (Figure 1A, bottom panel, Data S1). Each TF interacts with its own FLT3-ITD-specific gene module of downstream target genes. These modules can be AML specific, shared, or specific only to normal cells. To allow users to work with our data, we provide a link to GitHub (see STAR Methods) with the script together with all the data used in this manuscript and in Assi et al. to allow users to do the filtering themselves and combine our data with other data.

The majority of FLT3-ITD mutations co-occur with the NPM1 mutation,^12,13^ which was also true for our patient cohort (Table S1). We have previously shown that FLT3-ITD and FLT3-ITD/NPM1 cluster together^2^ and form very similar AML-specific regulatory connections between TF genes (Figure S1A). We therefore merged the data from both mutant groups and constructed a shared GRN. The shared FLT3-ITD-specific TF network is shown in Figure 1B. To test the robustness of our GRN construction, we determined the complete FLT3-ITD+ AML-specific GRN compared to healthy CD34^+^ mobilized peripheral blood stem cells (PBSCs) for each of nine patients, as outlined in Figure S1B, and we measured the number of FLT3-ITD+ AML-specific edges found in each patient network but not in healthy cells. Figure 1B shows the percentage of FLT3-ITD+ AML-specific edges between TF genes shared between patients, while Figure S1C shows in addition which edges are patient specific. The result of this analysis demonstrates that the specific connections are shared between more that 50% of all patients. The shared core FLT3-ITD+ AML-specific GRN shows multiple highly connected nodes containing FLT3-ITD+ AML specifically upregulated TF genes such as the KLF, RUNX, C/EBP, and FOX families and NFIX, all displaying multiple edges being connected to other TF encoding genes. This analysis shows that bound RUNX, ETS, and AP-1 sites occupy a central position in the network with multiple edges going in and out. It also explains why their motifs were highly enriched in FLT3-ITD-specific DNaseI hypersensitive sites (DHSs).

### The refined FLT3-ITD+ AML-specific GRN predicts genes required for AML maintenance

**Figure 2.**
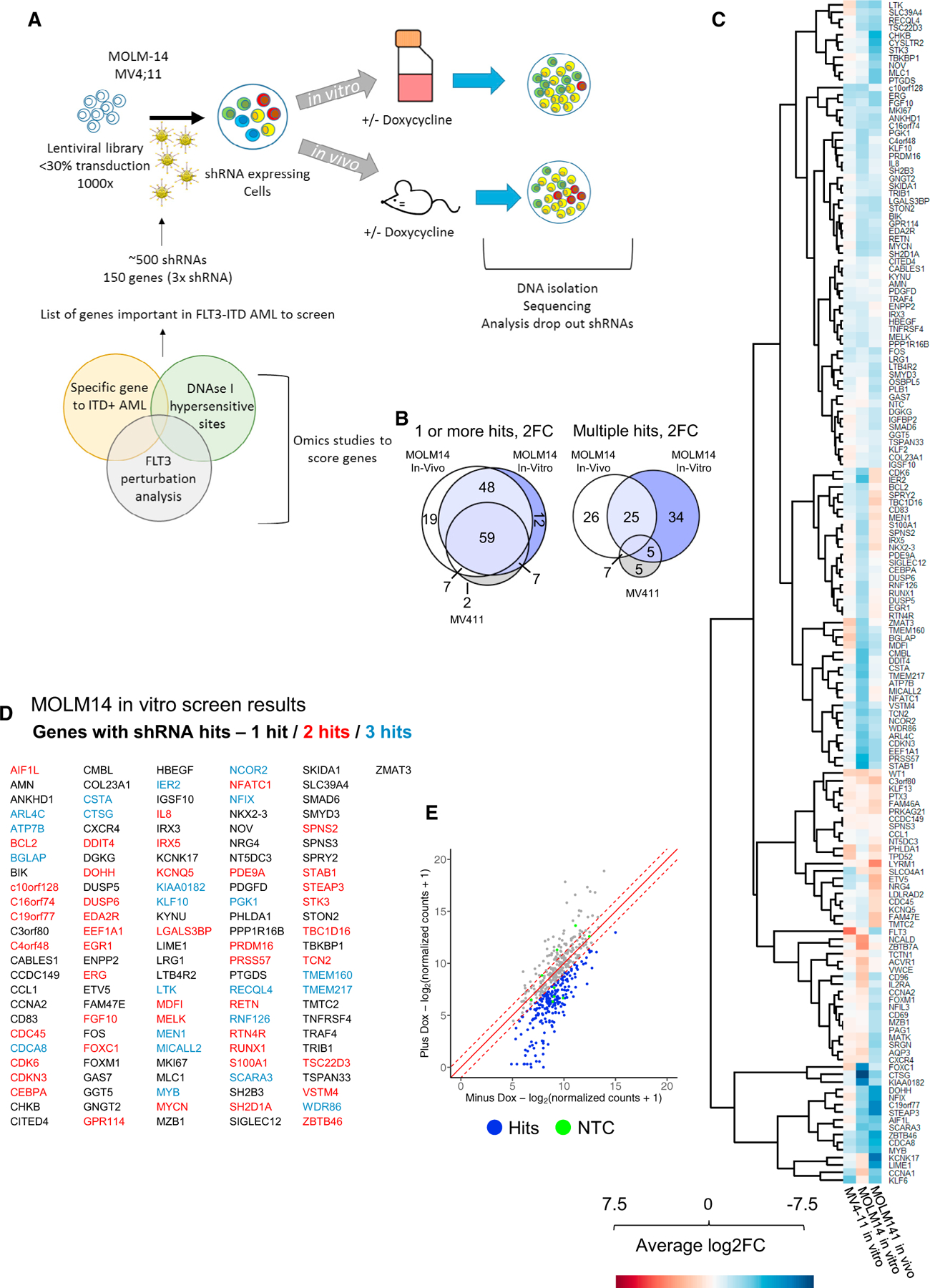
The GRN of FLT3-ITD and FLT3-ITD/NPM1 informs a highly efficient shRNAi screen (A) Scheme of shRNA screen in vitro and in vivo strategy. (B) Venn diagrams of genes with lost shRNAs in screen-2-fold change (FC), one or more hits-and genes with multiple hits showing a 2 FC in abundance. (C) Heatmap showing the average FC of shRNA abundance in three screens (average of all three shRNAs per gene) with the target genes plotted on the right. (D) MOLM14 in vitro screen results and list of genes with shRNA hits. (E) shRNA abundance after screen in MOLM14 in vitro plotted as scatterplot (further screens can be found in Figure S2).

We next tested the hypothesis that highly connected nodes within the refined GRN and some of their targets would be important for the maintenance of FLT3-ITD+ AML. To this end, we performed an informed shRNA screen targeting 161 genes selected from the FLT3-ITD GRN and the TF modules as described before^14^ (Figure 2A). The screen was performed in two FLT3-ITD+ cell lines (MV4-11, MOLM14) *in vitro* and for MOLM14 *in vivo* using immunodeficient NOD.Cg-Prkdcscid Il2rg tm1Wjl/SzJ(NSG) mice, as summarized in Figure 2A.

To identify a subset of genes important for FLT3-ITD/NPM1-mutated AML survival, we employed a rigorous filtering strategy (Figure S1D). Potential targets are listed in Figure S1E (Data S2) and were scored by (1) their specific RNA expression in FLT3-ITD+ AML vs. PBSCs and (2) whether DHSs linked to the gene by CHiC were specific to FLT3-ITD+ AML and (3) contained motifs relating to key nodes in the GRN. We also (4) included genes that were repressed after treatment with FLT3i in a PDX generated from a primary AML *in vitro* as measured by RNA-seq (Figure S1E, Data S3). Three shRNAs per target together with 10 non-targeting control shRNAs were designed and cloned into a doxycycline (DOX)-inducible lentiviral vector expressing a constitutive Venus and an inducible dTomato fluorochrome (Figure S1F). In our previous studies, we had already validated the importance of FOXC1, NFIX, and the AP-1 families represented by *FOS*,^2^ which were included as positive controls. Our targeted depletion screen showed a significant overlap between targets (Figure 2B) and, importantly, a very high hit rate, with the majority of selected targets being depleted, and nearly 50% of them being important for growth *in vitro* and *in vivo* (Figures 2B–2E and S2A–S2D) (Data S2). We compared our hits with those from the DepMap project (DepMap.org), which has tested the same FLT3-ITD+ cell lines in a genome-wide CRISPR screen. The majority of overlapping DepMap hits that have a gene effect of greater than −0.3 are also hits in our shRNA screen. Genes depleted in our screen and DepMap included multiple TF genes such as *EGR1*, *NFIL-3*, *FOS*, *RUNX1*, *IRX3*, *MYB*, *NFiX*, *and CEBPA*, together with cell cycle regulators such as *CCNA2*, epigenetic regulators such as *MEN1*, and signaling proteins such as *DUSP6*. However, our targeted screen identified several additional hits such as *KLF2*, *IRX5*, *NFATC1*, *IL8*, and *FOXC1.* We selected shRNAs against the TF genes *NFIL3*, *RUNX1*, and *EGR1* to manually validate the results in the cell lines using colony assays of MOLM14 cells (Figures S2E–S2G). We also expressed a dominant-negative version of C/EBP^15^ from a DOX-inducible plasmid to highlight the importance of this TF family for FLT3-ITD+ AML colony-forming ability (Figure S2H). Finally, we validated our results in primary cells and performed a smaller screen testing the non-targeting control (NTC), *NFATC1*, *NFiX*, *EGR1*, and *RUNX1* on ITD-15 PDX primary cells in culture (Figure S2I). Our larger screen also identified the dual-specificity phosphatases DUSP5 and DUSP6 as hits. To validate a non-TF target, we treated various cell lines representing different AML subtypes with the DUSP inhibitor BCI (Figure S2J) and showed that it is an efficient inhibitor of cell growth, albeit not in a FLT3-ITD+ AML-specific fashion.

Taken together, our experiments demonstrate that constructing a disease-specific GRN based on primary AML cell data is highly predictive for identifying highly connected network nodes required for the growth of FLT3-ITD+ AML cells.

### Identification of FLT3-ITD-specific overlapping transcription factor modules

**Figure 3.**
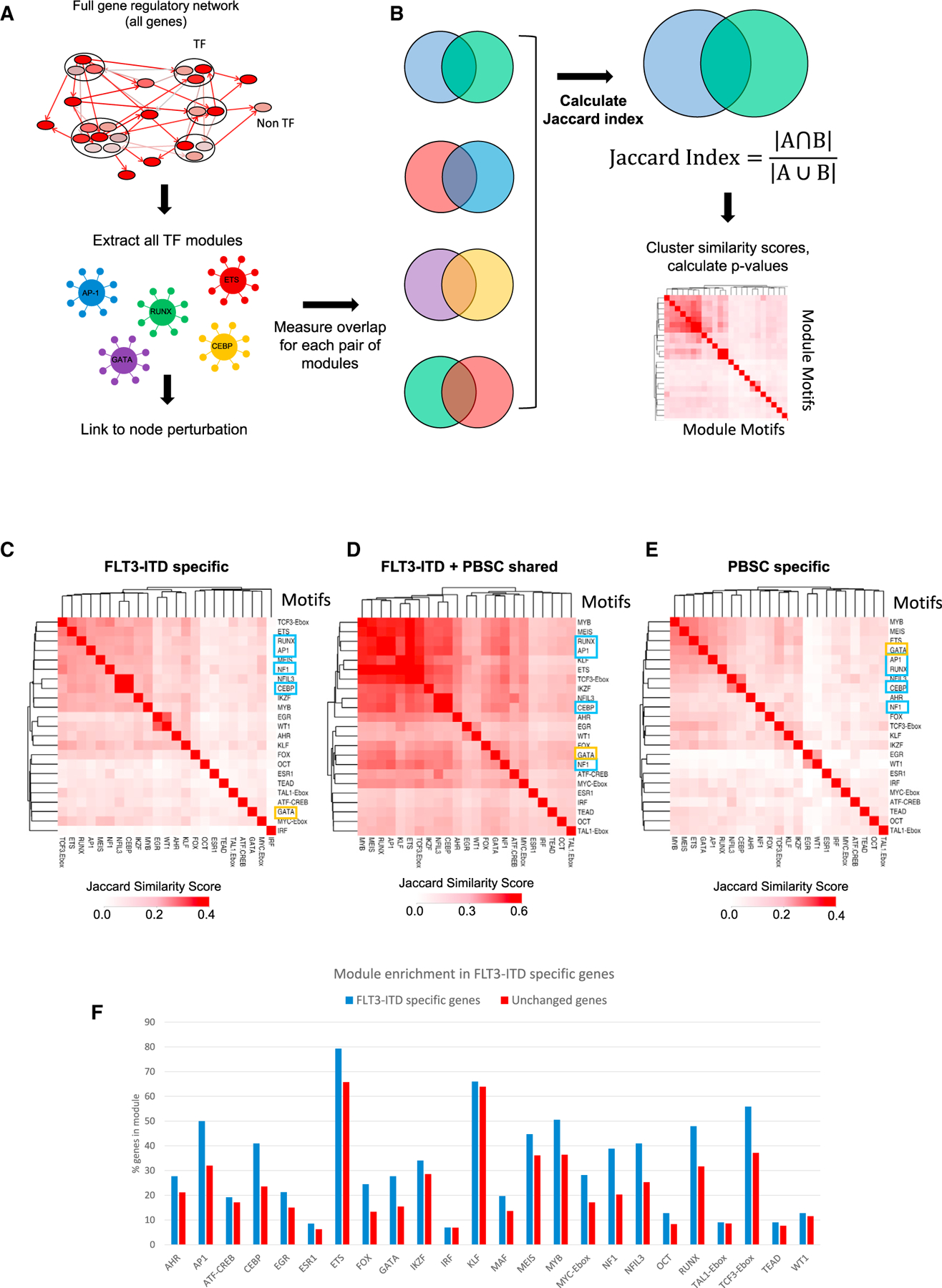
Identification and comparison of regulatory TF modules (A) Scheme of module identification. (B) cheme of identifying Jaccard similarity of TF regulatory modules in FLT3-ITD-specific DHSs compared to PBSCs. (C) Jaccard similarity of TF regulatory modules in FLT3-ITD-specific DHSs compared to PBSCs. (D) Jaccard similarity of TF regulatory modules in DHSs shared with PBSCs. Blue boxes: TFs whose module was perturbed in this study. Yellow box: GATA motifs. (E) Jaccard similarity of TF regulatory modules in PBSC-specific DHSs compared to FLT3-ITD. (F) Histogram showing enrichment of modules in FLT3-ITD in sites linked to upregulated mRNAs compared to PBSCs. Control genes are genes showing the same level of mRNA expression in PBSCs and FLT3-ITD+ AML. The y axis shows the percentage of genes in each group that are assigned to a TF family module.

Since TFs and other regulators of gene expression are part of an interacting network, targeting such molecules in a clinical setting requires careful examination of the effects of perturbation on their downstream targets and how GRNs shift in the absence of specific TFs. We therefore connected the above-described TFs to the wider patient-derived GRN by using digital footprinting and (where available) ChIP analyses to identify AML-specific and shared TF modules. The idea behind this analysis is to identify regulons (TFs and their targets) that we do not find in healthy cells. These analyses were conducted for individual TFs (C/EBP, RUNX, EGR) and the previously studied TF families (AP-1, FOXC1, NFIX) that were linked to downstream genes specifically upregulated in FLT3-ITD/NPM1 cells as outlined in Figure 3A. We also included occupied binding sites shared with normal cells as they often contain motifs for signaling-responsive TFs that could stimulate AML-specific gene expression in an aberrant signaling environment. Figure S3A and Data S4 show that each TF is connected to a large number of upregulated target genes, many of which are shared between different modules as exemplified by *DUSP5/DUSP6* or *WT1* (highlighted), suggesting that such genes are regulated by more than one factor. We also observed cross-regulation between modules as exemplified by FOXC1 being part of the NFI module (high-lighted). Note that FOXC1 and NFIX are examples of TFs that are aberrantly expressed compared to healthy cells.^2,16^ FOXC1 is truly mis-expressed, and NFIX plays a role in hematopoiesis and hematopoietic stem cell survival^17,18^ and is then downregulated, but in FLT3-ITD+ AML cells, it is strongly upregulated compared to healthy PBSCs. Their expression is therefore a part of the aberrant FLT3-ITD/NPM1 leukemic phenotype.

To examine the degree by which nodes are shared between AML and normal cells, we calculated their similarities as shown in Figure 3B. This analysis showed that the FLT3-ITD-AML-specific GRN (Figure 3C), which is distinct from that of healthy PBSCs, contains a central cluster of overlapping nodes for the TF modules TCF3-Ebox, ETS, RUNX, AP-1, MEIS, NFI, C/EBP/NFIL3, IKFZ, and MYB that is also found in the shared sites (Figure 3D). The healthy PBSC-specific central module cluster (Figure 3E) contained similar motifs but is characterized by the additional presence of GATA motifs indicating a more immature state of cells. We next determined all modules of the whole FLT3-ITD+ AML GRN and asked which ones were enriched in FLT3-ITD+ AML-specific genes (Figure 3F). This analysis showed again that specific modules were overrepresented in FLT3-ITD+ AML specifically expressed genes, including again AP-1, C/EBP, NFI, and RUNX1 modules.

Taken together, this analysis shows that FLT3-ITD+ AML-specific genes are regulated by distinct and overlapping sets of TF modules.

### Perturbation of FLT3-ITD+ AML-specific TF modules in primary cells highlights regulatory relationships based on combinatorial TF action

To examine, how the perturbation of FLT3-ITD+ AML-specific modules that are not shared with healthy cells would affect the viability of primary FLT3-ITD+ AML cells, we examined the crosstalk between selected TF modules by genome-wide analyses. To this end, we interrogated the RUNX1 and NFIX modules by shRNA and the larger C/EBP and AP-1 families by expressing their dominant-negative counterparts in primary PDX cells, followed by conducting an assay for transposase-accessible chromatin with sequencing (ATAC-seq) and RNA-seq (Figures S4B–S4F). As controls, we tested these targets together with shRNA against *DUSP5*, *FOXC1*, and *EGR1* in healthy CD34^+^ PBSCs by transducing a mini-library of these lentivirally expressed shRNAs together with an NTC, where no suppression of growth was observed (Figure S4A), indicating that perturbation had no or little effect on the viability of healthy cells. However, note that we cannot exclude an impact on smaller sub-populations of leukemic cells. In addition, we transduced FLT3-ITD+ AML cells with vectors expressing DOX-inducible dominant-negative peptides targeting the AP-1 and dnC/EBP TF families followed by a colony assay (Figures S4B and S4C). Again, the inactivation of these TFs had a profound effect on the growth of FLT3-ITD+ AML. In contrast, targeting DUSP5 and −6 phosphatase activity with inhibitor (E)-2-benzylidene-3-(cyclohexylamino)-2,3-dihydro-1H-inden-1-one (BCI) affected the growth of both FLT3-ITD+ AML and healthy PBCSs, albeit with different efficiency, as shown by inhibitor experiments (Figure S4D), suggesting that there may be a therapeutic window.

**Figure 4.**
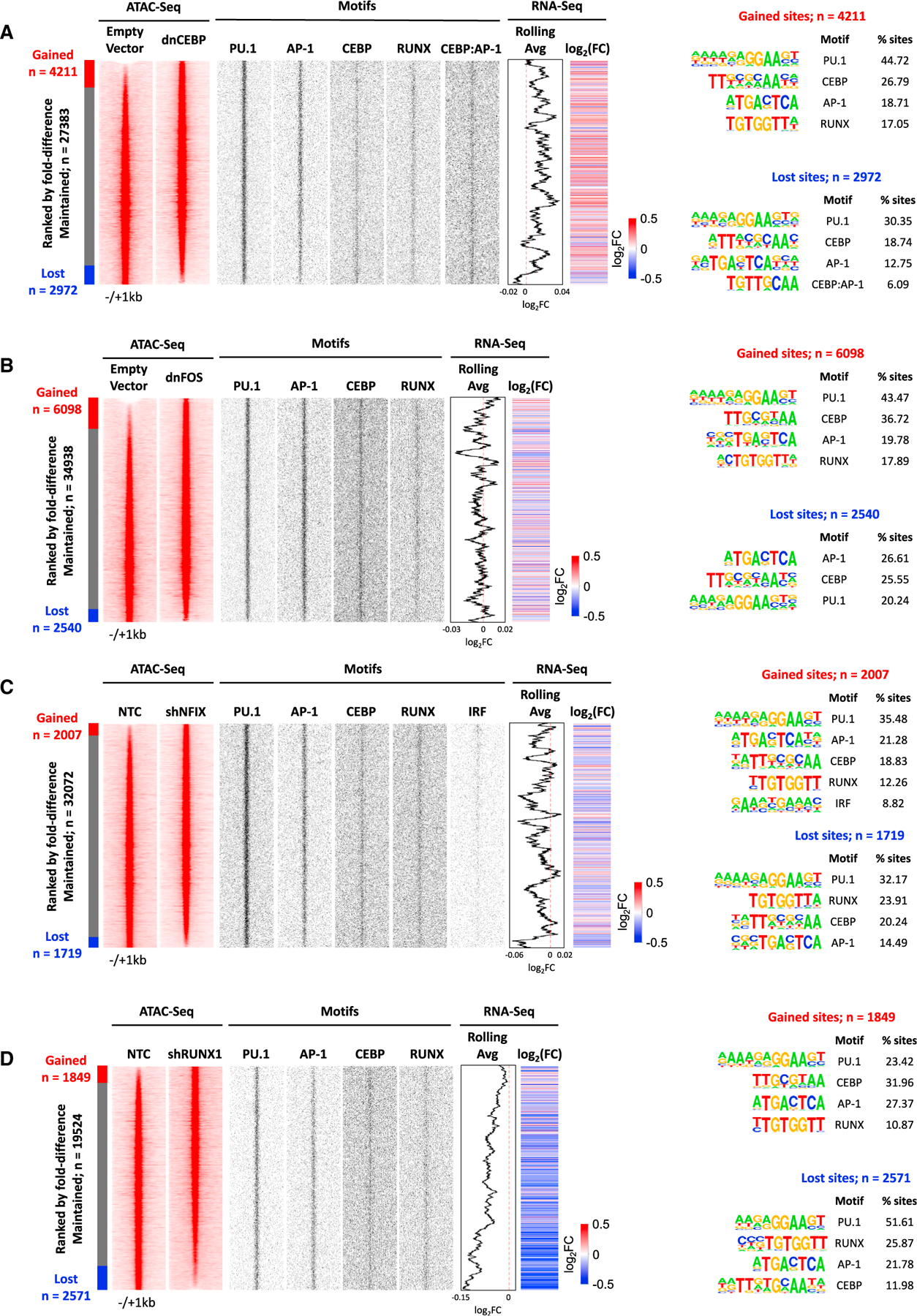
Perturbation of TF regulatory modules alters the chromatin landscape of FLT3-ITD primary AML cells (A–D) Density plots showing ATAC sites (left) changed after (A) dnCEBP, (B) dnFOS, (C) shNFIX, or (D) shRUNX1 expression compared to an empty vector control (A, B) or shNTC (C, D). Data are ranked by normalized tag counts of ATAC peaks from control cells over peaks obtained from transduced cells. Numbers of gained (red) and lost (blue) open chromatin regions are indicated to the left of the bar. Middle panels: TF binding motifs projected on hypersensitive sites are plotted alongside. Rolling average of gene expression values and FC of gene expression are plotted alongside the DHS (see also Figure S4). Right panel: enriched TF motifs in lost and gained open chromatin regions.

The analyses of ATAC-seq data showed that each perturbation had a strong effect on the chromatin landscape with thousands of sites opening and closing (2 FC), indicating that factor perturbation not only affected growth, but it also changed the GRN of the cells (Figures 4A–4D). However, despite sites being gained or lost, with one exemption, which is described below, the enriched motif compositions in those sites did not significantly change, with PU.1, RUNX, C/EBP, and AP-1 motifs dominating the picture, suggesting that the system rewires using the same TF modules.

**Figure 5.**
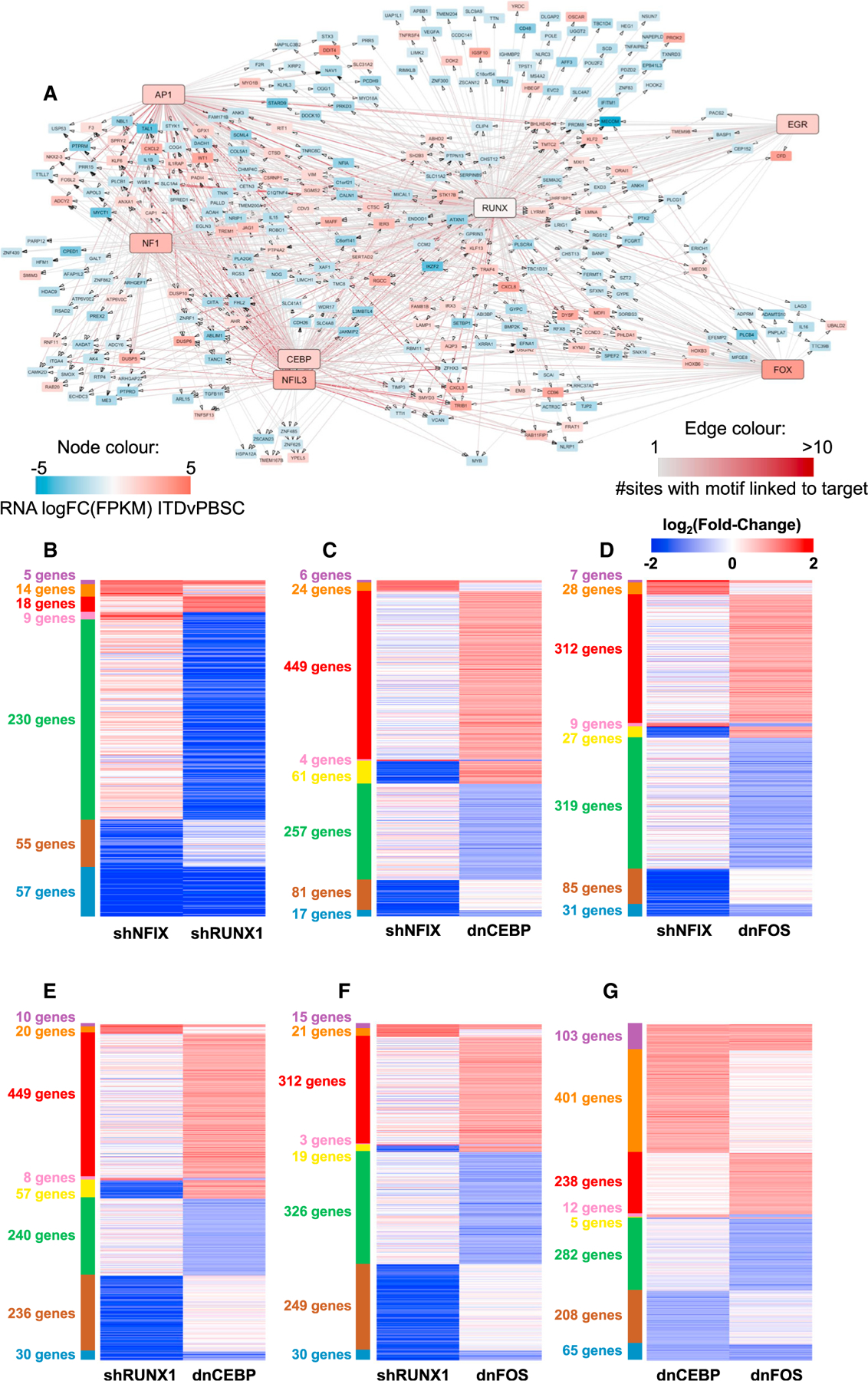
Perturbation assays reveal crosstalk between regulatory modules (A) Network showing the connection of the RUNX1 module to other indicated modules for genes upregulated (red) or downregulated (blue) compared to PBSCs. Node color indicates the FC in RNA expression of the gene in FLT3-ITD+ AML compared to healthy PBSCs. Edges indicate the presence of DHSs assigned to the gene-containing motifs, and edge color corresponds to the number of interacting DHSs. (B–G) Heatmaps showing pairwise comparisons of the log2FC RNA (CPM) of differentially expressed genes in primary FLT3-ITD+ AML cells (ITD-12) subjected to the indicated TF knockdowns compared to an empty vector control (dnCEBP, dnFOS) or shNTC (shRUNX1, shNFIX), respectively (see also Data S6).

The analyses of gene expression after factor perturbation showed a complex regulatory relationship between the modules (Figure 5, Data S5). All TF perturbations led to both an up- and downregulation of genes. Inspection of genes that responded to NFIX and RUNX1 knockdown revealed that NFIX is in the same regulatory pathway as RUNX1, whereby *RUNX1* is strongly downregulated after NFIX knockdown together with 56 other genes. RUNX1 depletion affected direct RUNX1 target loci driving macrophage differentiation such as *CSF1R* and *IRF8*^19,20^ and multiple inflammatory genes (Data S6). The indirect effects of RUNX1 downregulation can also be seen in the NFIX knockdown, although the FC of many genes did not reach the significance level. Another cross-module response is seen after dnFOS expression, which downregulates NFIX and its homolog NFIA. It has been shown that AP-1 (JUN) and C/EBP family members can physically interact to drive macrophage differentiation,^21^ and our data show them to co-regulate a number of genes. In this context, it is interesting to note that one the motifs enriched in open chromatin regions lost after dnC/EBP expression is a C/EBP/AP-1 composite motif (Figure 4A), suggesting that the two factors indeed cooperate.

Taken together, these few examples indicate an extensive crosstalk between the different regulatory modules. Perturbation of one module leads to a complex response of other modules.

### RUNX1 is an essential factor for the establishment of a FLT3-ITD-specific gene expression program and is involved in cell-cycle regulation

Our experiments show that the perturbation of each selected TF module from the AML-specific GRN led to an abolition of AML colony-forming ability and growth. To address the molecular basis of this finding, we concentrated on the RUNX1 module. Various AML subtypes, in particular core binding factor AMLs,^22–24^ are dependent on the presence of a wild-type copy of RUNX1. In addition, the analysis of leukemia reconstituting cells derived from induced pluripotent stem cells from patients with FLT3-ITD/NPM1 suggested an important role for this TF in this AML subtype as well.^25^ To ensure that our digital footprint-based module construction was valid, we validated binding sites by comparing them to previous ChIP-seq experiments in patients with FLT3-ITD/NPM1 and cell lines^1^ (Figures S5A and S5B) and found that 79% of genes within the module are RUNX1 ChIP targets (Figure S5B), and 55% of all footprinted sites (Figure S5C) are also bound by RUNX1. In FLT3-ITD+ AML, the RUNX1 module strongly interconnects with the other modules (Figure 5A) to establish a FLT3-ITD-specific gene expression pattern, and its motifs are the most enriched (note the co-enrichment of NFI motifs) (Figure S5D). The importance of RUNX1 in establishing this gene expression pattern is high-lighted by the fact that in a patient with FLT3-ITD where one RUNX1 allele is mutated (F131fs), it is abolished (Figures S5E–S5G), and FLT3-ITD specific genes expression is suppressed (Figure S5H). Moreover, as shown before,^1,2^ FLT3-ITD specifically expressed genes are highly enriched in the RUNX1 module (Figure S5I).

**Figure 6.**
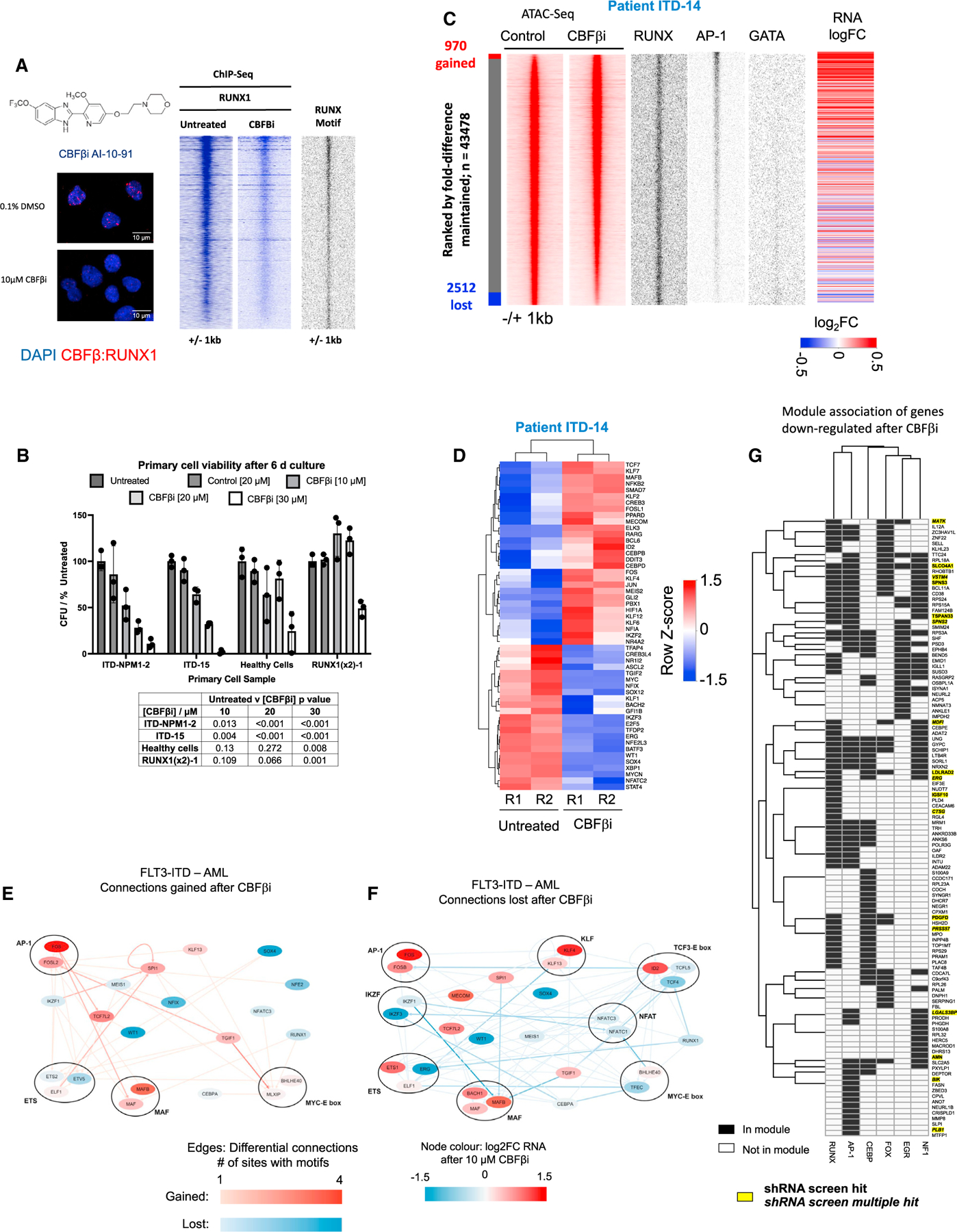
The RUNX1 regulatory module is essential for FLT3-ITD-specific gene expression: dissection of its contribution (A) Structure of CBFβi and representative images of proximity ligation assay of primary FLT3-ITD+ AML cells (ITD-14) with and without 10 μM CBFβi treatment, where blue signal shows DAPI nuclear staining, and red signal indicates interactions between CBFβ and RUNX1; scale bar represents 10 μm (see also Figure S6). Right panel: density plots of ChIP experiments showing the genome-wide signal of RUNX1 binding with and without CBFβi, as well as RUNX motifs present at the binding sites plotted alongside. (B) Viability assays with the indicated primary cell types treated with increasing concentrations of CBFβi. RUNX1 (2x): cells from a patient with a double RUNX1 mutation. Error bars show standard deviation and p values (table) were calculated relative to untreated cells using Student’s t test (n = 3). (C) Density plots of ATAC-seq analysis (red) of primary FLT3-ITD+ patient cells (ITD-14) with and without 10 μM CBFβi ranked against each other according to FC with the indicated TF motifs (black) at the open chromatin sites and the logFC expression of the associated genes present plotted alongside. (D) Unsupervised clustering of transcription factor gene expression of primary FLT3-ITD+ patient cells with and without CBFβi. (E and F) Gained (E) and lost (F) connections in the FLT3-ITD-specific GRN before and after CBFβi treatment. Red edges show gained connections after CBFβi, with blue showing those that are lost, and node color represents the FC in RNA expression of the TF after CBFβi. (G) Genes downregulated in the RNA-seq data in two or more of the CBFβi-treated patients. The heatmap shows the gene modules associated with each gene (black = associated, white = not associated). Yellow highlight: genes scoring in our screen. Single hits in the screen in one or more samples are in written in bold; if there were multiple hits in one or more samples, they are in italics. Genes not in the RUNX1, AP1, CEBP, EGR1, FOX, or NF1 module are not included in this data. Hierarchical clustering was performed to group genes into similar modules.

The fact that the depletion of highly connected TF targets within AML-specific GRNs affects AML but not normal cells gives rise to the hope that malignant epigenetic states can be reprogrammed, and cells can be driven into a cellular state that is incompatible with survival. TFs were thought to be “undruggable,” but in recent years, significant progress has been made to target these factors.^26^ We therefore probed the FLT3-ITD-specific GRN with the small molecule AI-10-91 (CBFβi), which disrupts the interaction between the RUNX DNA-binding domain and its binding partner CBFβ.^27^ We first used a proximity ligation assay to determine the optimal time point to detect a complete dissociation of RUNX1CBFβi (Figures 6A and S6A), which we found to be 8 h, suggesting that the complex is quite stable within the cell. ChIP experiments with primary cells treated with CBFβI confirmed a widespread loss of RUNX1 from the genome (Figure 6A, right panel). The inhibitor had a profound effect on the viability and colony-forming ability of FLT3-ITD/NPM1 and FLT3-ITD+ AML cells but not healthy cells in culture (Figures 6B and S6B). The CBFβ inhibitor efficiently killed most cells after 6 days. Furthermore, the cells from a patient with a double RUNX1 mutation (RUNX1(2x)) did not respond to the inhibitor except when a very high dose was used, again demonstrating that inhibition operates via the RUNX1 module.

Similar to the depletion of RUNX1 by shRNA-mediated knockdown, the removal of RUNX1 from the genome led to not just a loss but also the gain of open chromatin regions and changes in gene expression (Figures 6C and S6C, left panel). Again, inhibition yielded a complex response, with the expression of numerous FLT3-ITD/NPM1-specific genes in three different patients being up- or downregulated in similar patterns (Figure S6D, Data S7) including multiple genes from the RUNX1 module (Figure S6D, right panel, Data S7). RUNX1 inhibition changed the expression of multiple TF genes (Figure 6D), suggesting that alongside the effect of CBFβi on cellular growth, the GRN was rewired in response to treatment. We confirmed this idea using our ATAC-seq data, and we constructed GRNs from open chromatin sites that were gained and sites that were lost after inhibitor treatment (Figures 6E and 6F). The analysis of lost edges (Figure 6F) showed that multiple connections to the RUNX1 and to other TF nodes, such as the *CEBP* node, were lost, but other connections such as from and to the AP-1 family were gained (Figure 6E), indicating that not only connections to RUNX1 but also to other TFs were altered. To close the circle, we therefore asked how CBFβi treatment affected the genes belonging to the other five modules (NFI, AP-1, FOX, EGR, C/EBP) and how these associations were reflected in our shRNA screen (Figures 6G and S6E). The analysis of genes downregulated after treatment (Figure 6G) showed that the inhibition of RUNX1 activity had a profound influence on genes organized in the other modules, even when they were not overlapping with the RUNX1 module. An example for this finding is *PLB1*, which encodes for phospholipase B1, whose overexpression has been observed in glioblastoma and has been highlighted as an mRNA vaccine candidate (Figure 6G).^28^ Down-regulated genes from other modules overlapping with the RUNX1 module scoring in our screen included MATK (megakaryocyte-associated tyrosine kinase), which is an important regulator of SRC kinases in blood cells.^29^ The analysis of upregulated genes after CBFβi showed a strong enrichment of genes organized in the overlapping NFI, AP-1, and RUNX modules scoring in our screen (Figure S6E). This included again *DUSP6* but also the genes encoding the Zn^2+^ finger TFs KLF2 and KLF6, which have been shown to be repressed in AML cells and which are associated with myeloid differentiation.^30^

**Figure 7.**
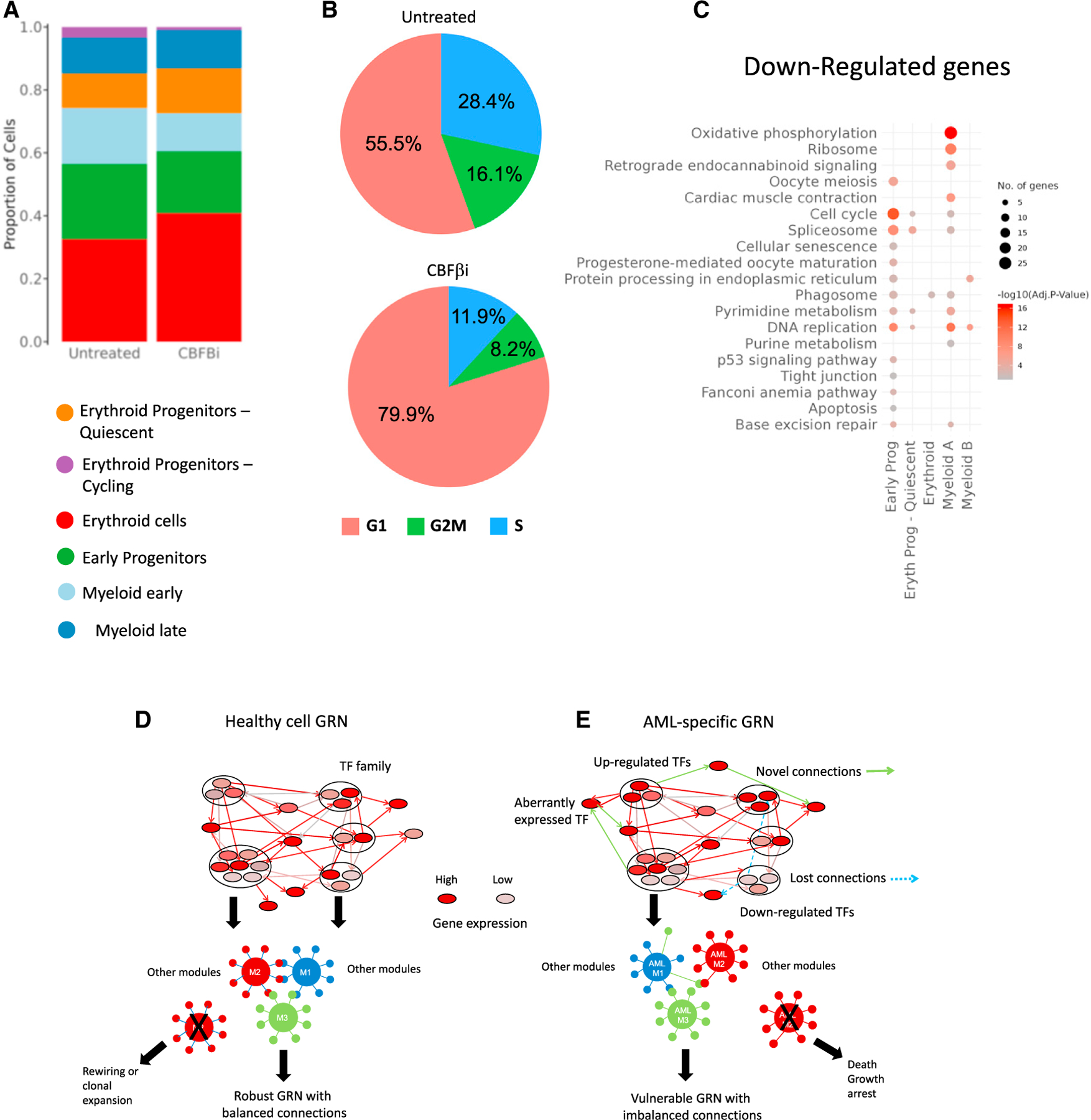
RUNX1 inactivation impedes cell-cycle progression in blood progenitors (A) Bar plot showing the proportion of the indicated cell types in the population with and without CBFβi treatment. (B) Pie chart showing the proportion of cells in the indicated cell-cycle phase without (upper panel) and with (lower panel) CBFβi treatment. (C) Gene Ontology terms of genes downregulated after CBFβi treatment. (D and E) Model describing the difference between a robust healthy GRN in blood progenitors (D) and a vulnerable GRN found in malignant cells (E).

In our final set of experiments, we asked the question of which cell type in primary AML cells and which genes were most affected by CBFβi. To this end, we cultured primary FLT3-ITD+ AML cells in the presence and absence of CBFβi and conducted single-cell (sc)RNA-seq experiments. Untreated cultures contained a mixture of early precursor cells and myeloid and erythroid cells of variable differentiation stages (Figures 7A and S7A; see S7B and S7C for a differentiation trajectory analysis). Inhibitor treatment reduced cell numbers with early precursors with myeloid cells being the most affected cell types (Figure 7A, right panel). The analysis of cell cycle specifically expressed genes before and after inhibition showed a block in G1 that affected all cell types (Figures 7B, S7E, and S7F). The inspection of downregulated genes in early progenitors revealed a strong downregulation of cell-cycle regulators and ribosomal genes (Figure 7C), which was consistent with the cell-cycle block. Also in this experiment, the cell-cycle block was followed by cell death as measured by a viability dye assay.

In summary, our experiments show that targeting an important TF comprising a highly connected node within an AML-subtype-specific GRN such as RUNX1 leads to a profound impact on other TF modules, which rewires the GRN of AML cells, leading to a cell-cycle block and, eventually, cell death.

## DISCUSSION

The present study shows how identification of an aberrant AML-subtype-specific GRN based on the data collected by Assi et al.^2^ leads to the identification of genes whose expression is vital for the maintenance of malignant cells. The hit rate for our screen was very high, and we found numerous TF-encoding genes that were important for the growth of AML but not normal cells. Moreover, the identification of the downstream targets of such TFs highlighted a complex web of interactions between genes that were essential for FLT3-ITD/NPM1 AML growth. This and our previous studies show that the idea of an AML-subtype-specific GRN that maintains AML cells is not only true for groups affected by mutations of gene regulatory molecules themselves but also for those with signaling mutations who generate chronic growth signals, such as the FLT3-ITD. Decades of clinical trials have tried to target aberrant signaling and have failed due to the development of drug resistance. Moreover, such treatments only affected fast-growing cells and not quiescent leukemic stem cells. Based on these data and those of others,^31,32^ we believe that the most viable way to eliminate malignant cells is to identify the factors crucial for tumor maintenance and reprogram cells into either normal cells that differentiate or change their GRNs into an unstable, non-viable state by identifying TFs that are essential for AML maintenance.

Our GRN analyses inform us which factors and genes to target and which factors when targeted do not or only minimally/temporarily affect healthy cells. As schematically outlined in Figure 7D, in healthy cells, after hundreds of millions of years of evolution of complex multicellular organisms, the balance between self-renewal, differentiation, and growth is highly robust, and cells do not rely on one factor alone for differentiation, as exemplified by non-malignant clonal hematopoiesis. Moreover, as seen in the similarity of modules in the GRN shared between aberrant and normal cells (Figure 3D), all modules are in balance, and all interact with each other (note the high cross-module similarity and the high number of connections between modules), thus creating a stable network. With the possible exception of MLL translocations, which involve global activators of gene expression, it takes several mutations to derail normal hematopoietic differentiation with the majority of initiating mutations causing AML being members of the gene regulatory machinery. Mutant cells compensate for the loss or impediment of such activities by rewiring their GRNs, thus becoming dependent on alternative factors and pathways, which are often different from those of normal cells (Figure 7E). Mechanisms of this kind are often the upregulation of alternate signaling pathways, overexpression of TF genes, or the ectopic activation of TF genes normally not expressed in these cells. This phenomenon can also be seen in the FLT3-ITD subtype whose AML-specific GRN highlights various TF families that are associated with aberrant signaling (AP-1 family), are overexpressed (RUNX1),^1,33^ or are aberrantly expressed (FOXC1, NFIX).^2,16^ In addition, these subtype-specific deregulated TFs form regulatory modules that contain multiple signaling genes that are upregulated compared to healthy cells, such as the *DUSPs* or TNF superfamily members (see Figures 5A–5F). Genes are regulated by a multitude of TFs that form interacting protein complexes on enhancers and promoters.^34,35^ Such interactions can be aberrant as exemplified by the formation of AML-specific protein complexes containing FOXC1 and RUNX1.^36^

Recent experiments coupling degrons to oncoproteins or normal TFs have shown that only a small number of genes immediately respond to the degradation of one factor, and that it takes a number of hours until the whole GRN responds and changes the expression of multiple genes.^37^ It takes even longer until cells differentiate or die, but it is this feature that is relevant in a clinical setting. Our data highlight the molecular reason for this finding by showing that an AML-specific GRN sustaining leukemic growth is stabilized by multiple modules that cooperate in driving AML-subtype-specific expression. The removal of one essential module leads to a rewiring of others, as seen in our detailed studies of the RUNX1 module. Here, the result is a profound cell-cycle block followed by eventual cell death, suggesting an inability of RUNX1-depleted cells to further rewire their GRN into a stable state which could be the basis of therapy. A recent study applying a degron technology to target IKZF2 in MLL-driven AML^38^ demonstrates that such a strategy is indeed feasible.

### Limitations of the study

We are aware that there are several limitations to the study. (1) Although the basic data informing the screen were obtained from patients with AML, for technical reasons, our screen had to be performed in cell lines. It was not possible to validate all hits in primary cells, including hits that may also affect healthy cells, and we had to decide which ones to validate. However, our perturbation studies still provide an important resource for follow-up experiments. (2) We have not filtered the FLT3-ITD+ AML GRN subtracted from that of healthy cells against the GRNs of other AML subtypes, and we have not performed a screen based on data filtered this way. Such an approach would highlight targets exquisitely specific for FLT3-ITD+ AML but would have missed pan-AML targets, and for this reason, we decided against it. However, to allow such studies, we provide a GitHub link that enables the community to perform their own GRN filtering. (3) It is possible that the xenotransplantation experiments do not fully reflect the human situation. Human bone marrow organoids were recently described, which should alleviate this problem.

## STAR★METHODS

### RESOURCE AVAILABILITY

#### Lead contact

Further information and requests for resources and reagents should be directed to and will be fulfilled by the lead contact, Constanze Bonifer (c.bonifer@bham.ac.uk).

#### Materials availability

Plasmids generated in this study are available upon request from the lead contact.

#### Data and code availability

- All sequencing data produced as part of this study are available on GEO under the super series GSE236775.
- Python scripts used to construct the gene regulatory networks presented in this study, as well as the probability weight matrices for the transcription factor binding motifs and promoter-capture HiC data have been made available on GitHub at https://github.com/petebio/Gene_regulatory_network_analysis and are free to use under an MIT license, https://doi.org/10.5072/zenodo.268.
- Any additional information required to reanalyse the data reported in this paper is available from the lead contact upon request.

### EXPERIMENTAL MODEL AND STUDY PARTICIPANT DETAILS

#### Primary sample and PBSC processing

Human tissue was obtained with the required ethical approval from the National Health Service (NHS) National Research Ethics Committee. AML and PBSC samples used in this study were either surplus diagnostic samples or fresh samples obtained with specific consent from the subjects. AML samples were obtained from the Center for Clinical Haematology, Queen Elizabeth Hospital Birmingham, Birmingham, UK, or the West Midlands Regional Genetics Laboratory, Birmingham Women’s NHS Foundation Trust, Birmingham, UK. Mononuclear cells were purified on the same day they were received, and in most cases were also directly further purified using either CD34 or CD117 (KIT) magnetic antibodies as described.^1^ For some samples with >92% blast cells, column purification was not performed. Mobilized PBSCs were provided by NHS Blood & Transplant, Leeds, UK, and NHS Blood & Transplant, Birmingham, UK.

Primary human AML blast cells were cultured on primary human mesenchymal stem cells (hMSCs). Primary hMSCs from “normal” bone marrow were cultured in alpha-MEM (Lonza) supplemented with 10% fetal calf serum (Gibco) and 2 mM L-Glutamine (Gibco). 24 h prior to addition of primary AML cells hMSCs were seeded at 5000 cells/cm^2^ in tissue culture plates pre-treated for 20 min with 0.1% glycine.

Primary human AML blasts were defrosted and cultured at 0.3–0.5 x 10^6^ cells/ml on hMSC feeders in alpha-MEM (Lonza) supplemented with 12.5% fetal calf serum, 12.5% horse serum, 100 U/ml penicillin/streptomycin, 2 mM L-Glutamine (all Gibco), 1 μM hydrocortisone (Merck) and 57.2 μM β-mercaptoethanol (Merck), 20 ng/mL IL-3, G-CSF and TPO (Pepro Tech) as described previously.^53^ Cells were passaged to new feeders every 7 days. All cells were cultured and treated in an incubator at 37C with 5% CO_2_.

#### Cell lines

MV4-11 (DMSZ, AC102), MOLM14 (DSMZ, ACC 777), Kasumi-1 (DMSZ, AC220), KG1a (DMSZ, ACC690) and P31/FUJ (JCRB Cell Bank, JCRB0091), SKNO-1 (DMSZ, ACC 690) were used in this study. All cell lines were cultured in RPMI 1640 supplemented with 1% L-glutamine and 20% heat-inactivated FBS, with 10 ng/mL GM-CSF (PeproTech) added to the SKNO-1 cultures. For culture maintenance cells were split to 0.5x10^6^ cells/ml every 3 days to not exceed 1–2 x 10^6^ cells/ml. For the *in vitro* screen after sorting the media was also supplemented with 1% Penicillin/Streptomycin. HEK293T cells (DSMZ, ACC305) were used to produce lentivirus. These cells are cultured in HEPES-modified DMEM medium supplemented with 10% FBS, 4mM L-glutamine and 1mM sodium pyruvate. Cells were split using trypsin every 3 days to not exceed a confluency of 70%. All cells were cultured and treated in an incubator at 37C with 5% CO2.

#### Mouse studies and PDX generation

All mouse studies were carried out in accordance with UK Animals (Scientific Procedures) Act, 1986 under project licence P74687DB5 following approval from Newcastle University animal ethical review body (AWERB). Mice were housed in specific pathogen free conditions in individually ventilated cages with sterile bedding, water and diet (Irradiated RM3 breeding diet, SDS Ltd). All procedures were performed aseptically in a laminar flow hood.

NSG mice (NOD.Cg-Prkdcscid Il2rg tm1Wjl/SzJ) aged between 12 and 16 weeks, both sexes, from an in-house colony were used for PDX generation. They were transplanted intra-femorally with 1x10^6^ patient or PDX cells under isoflurane anesthetic and administered with subcutaneous NSAID analgesia (5 mg/kg subcutaneous Carprofen).

Mice were checked daily, weighed and examined at least once weekly to ensure good health. Endpoints for humane killing were pale extremities, hunched posture, 20% weight loss compared to highest previous weight or 10% weight loss for 3 consecutive days.

PDX cells were harvested from spleen and isolated by passing through a 50μM cell sieve (Falcon Corning). Cells were washed in PBS and stored frozen in 10%DMSO/90%FBS.

For the *in vivo* shRNA screen 10 female mice Rag2^−/−^Il2rg ^−/−^ 1293Balb/c (RG) from an in-house colony and aged 8–10 weeks were injected intra-venously with 50.000 MOLM-14 cells containing the shRNA library per mouse in a volume of 100μL. Mice were randomly assigned to 2 groups. One group was fed the normal RM3 diet and one doxycycline containing diet (823747 − CRM (E) + 625ppm Doxycycline (P) 1kg 25kG, SDS Ltd) *ad libitum* on the day of cell injection. Diet was replaced every 3 days and mouse health assessed daily. Mice were humanely killed 19–22 days after cell injection when a weak tail or hind legs were first detected. Cells were isolated from spleen as above. Cells were isolated from the bone marrow by crushing the leg and hip bones in PBS in a pestle and mortar, vortexing and passing the supernatant through a cell sieve. Engrafted cells were sorted by FACS (Aria II, BD) using Venus and dTomato fluorophore DNA was isolated using the DNeasy Blood & Tissue Kit (Qiagen, 69504) following manufacturer’s instructions.

### METHOD DETAILS

#### FLT3-ITD AML shRNA screen

##### Vector

The vector used in this study (now named pL40C) is described in detail in.^54^ As described, the vector contains ampicillin resistance, a doxycycline-induced cassette that expresses the shRNA together with the fluorochrome dTomato and a constitutively expressed cassette containing the fluorochrome Venus.

##### shRNA oligo design

The shRNA oligos were designed using the informatic tool (https://felixfadams.shinyapps.io/miRN/) described previously.^54,55^ 161 genes were included as targets and 3 shRNA oligos were designed per gene as described in the text. As a positive control, FLT3 was included together with 10 NTC shRNA as negative controls. The oligos were ordered from Sigma Aldrich. Each oligo was 67 bp and was received with pre-mixed forward and reverse oligos at a concentration of 100 μM with desalt purification.

##### shRNA library cloning

The library of shRNA was produced following the process described in.^54^ Briefly, the oligos were phosphorylated and annealed. Afterward, all the oligos were pooled together. The vector was opened using the restriction enzyme BsmBI (Thermo, ER0451) following manufacturer recommendations. The opened plasmid was separated by running the product of the digestion in an agarose gel and extracting the DNA from the band using the Qiaquick Gel Extraction Kit (Qiagen, 28706) following manufacturer instructions. Plasmid and oligos were ligated using T4 DNA ligase kit (Thermo fisher, EL0011) with a molar ratio of 1:3 of vector and oligo. The ligation product was then transformed and amplified using XL-gold bacteria (Agilent, 200315) according to the manufacturer’s protocol. After the transformation a maxiprep was perform to obtain high amounts of the cloned library, we used the EndoFree Plasmid Maxi Kit (Qiagen, 12362) and followed manufacturer’s instructions.

##### Lentivirus production

The production of the lentivirus was done following the protocol described in Martinez et al.^14^ In summary, HEK293T cells were cultured in 15cm petri dishes up to a confluence of 50–60%. The library vectors and vectors for packaging and envelope (pMD2.G and pCMV∆R8.91) were mixed with special water (deionized water with 2.5 mM HEPES) and CaCl_2_ 0.5 M solution. Next, the mix of the plasmid/special water and CaCl_2_ was combined by dropwise addition of HeBS. After incubation the solution was poured dropwise into the plates for transfection of the HEK293T cells. After one day the media is change, following 48h of incubation the supernatant containing the virus is collected, spin and freeze.

##### Cell transduction

MV4-11 and MOLM-14 cells were transduced following the protocol described in Martinez et al.^14^ As a summary, cells at 10^6^ cells/ml were transduced with the pooled library shRNA lentivirus particles present in the supernatant using Polybrene at a final concentration of 8 μg/mL. Afterward, the plate was centrifuged at 34°C for 50 min at 900xg. After centrifugation, cells were incubated for 3 days. The transduction was performed at a low MOI (0.3 TU/cell) to produce a population of cells with one integration event per cell. Following lentiviral transduction, cells successfully carrying the fluorescent Venus constitutive expressed were purified using fluorescence-activated cell sorting (FACS) in an ARIA II. Most cells that remained after selection carried a single copy of the inducible shRNA. Cells were then used to perform the screening as described.

##### In vitro *screen*

For the *in vitro* screen we aimed for a coverage of 1000x of the library. Two different conditions were tested no doxycycline and doxycycline treatment. For transduced MOLM-14 the concentration of doxycycline (Sigma Aldrich, D5207) used was 500 ng/mL and 1μg/ml for transduced MV4-11. 5 million cells per condition were cultured at a concentration of 0.5x10^6^ cells/ml, split every 3 days to maintain that concentration, change the media and refresh the doxycycline. Cells were cultured for 15 passages and samples were collected at different time points. For obtaining DNA, cells were collected, centrifuged at 300 xG for 5 min and the pellet was frozen. The DNA was then isolated using the DNeasy Blood & Tissue Kit (Qiagen, 69504) following manufacturer’s instructions.

##### Library preparation for shRNA screens

PCR of genomic DNA was performed using ExTaq (Takara) using custom designed primers with Nextera i5 and i7 index sequences (Data S2) to amplify the mir30 insert containing the shRNA. Amplicons were electrophoresed on an agarose gel and DNA was purified using the QIAquick gel extraction kit (QIAGEN) according to manufacturer’s instructions and further purified by ampure (Beckman Coulter). Samples were pooled and analyzed on a Next Seq 2000 75 using a NextSeq 500/550 High output kit.

#### FLT3-ITD cell line validations

MV4-11 cells were transduced with lentiviral vectors and cultured as described above. For colony formation assays, MV4-11 cells were treated with 1 μg/mL doxycycline for 24 h prior to seeding at 5000 cells/ml in Methocult H4100 (StemCell Technologies) supplemented with Iscove’s MDM (Merck) and FCS (Gibco) at a 2:2:1 ratio. 1 μg/mL doxycyline was added to the media and colonies were counted after 8 days.

For Western blot analysis of protein expression, MV4-11 cells were cultured for 3 days with 1 μg/mL of doxycycline added every 48 h. 12 μg of protein extracts in Laemmli buffer were run on a 4–20% gradient pre-cast gel (Bio-Rad) and transferred to nitrocellulose using Turbo transfer packs (Bio-Rad). Membranes were blocked with 10% milk in TBS-T (10 mM Tris-HCl pH 7.5, 75 mM NaCl, 0.1% Tween 20) before being incubated at 4°C overnight in 5% milk TBS-T with primary antibody (αEGR1: 1:1000 (sc110 – SantaCruz), αNFIL3: 1:1000 (A302-606A, Bethyl), αRUNX1 (1:1000, 8758 cell signaling)). After washing in TBS-T, membranes were incubated in 5% milk TBS-T with HRP-conjugated anti-rabbit (Cell Signaling Technologies) or anti-goat antibody (Jackson ImmunoResearch) for 1 h at room temperature. After a further 3 washes in TBS-T, enhanced chemiluminescent reagent (Amersham) was applied and the blot was visualised using a GelDoc system (Bio-Rad). For loading controls, the membranes were stripped using Restore Stripping Buffer (Thermo Fisher Scientific) and GAPDH (ab8245; Abcam) and anti-mouse HRP linked antibody (Cell signaling Technologies) were applied and visualised as above.

#### Inhibitor experiments in primary AML cells and healthy cells

The DUSP 1/6 inhibitor BCI (Selleckchem) and FLT3-ITD inhibitor Quizartinib (Selleckchem) were dissolved to a 10 mM stock concentration in DMSO (Merck) on arrival. CBFβi (AI-14-91) and its control compound (AI-4-88)^27^ were both dissolved to a 40 mM concentration in DMSO.

Prior to dosing, primary cells were cultured as described above for 7 days after defrost. Samples were then transferred to a 96 well plate previously prepared with hMSC feeders and the desired concentration of inhibitor was added to the media (“untreated” control was treated with 0.1% DMSO). Cells were then incubated with the inhibitors for 6 days before viability was assessed by counting cells on a haemocytometer after a 1:1 dilution with Trypan Blue (Merck) to differentiate alive and dead cells. For dose-response curves IC50 was calculated using Graphpad prism software by performing non-linear regression (log[inhibitor] vs. normalized response).

For colony formation assays – cells were treated with the inhibitor for 24h prior to seeding at a density of 5000 cells/ml in Methocult Express (StemCell Technologies). The inhibitor was also added to the colony medium at the same concentration. Colonies were counted after 12 days.

For NGS experiments – primary cells were treated with the desired concentration of inhibitor for 24 h prior to harvest with 0.1% DMSO as a control.

#### Lenitviral transduction of primary AML cells and healthy cells

pL40c shRNA were generated by cloning shRNAs (Data S2) into the pL40c vector. The dnFOS and dnCEBP inserts, originally generated by Charles Vinson (National Cancer Institute, Bethesda, MD, USA), were cloned into a pENTR backbone and then Gateway cloning was used to insert that into the Tet-on plasmid pCW57.1 (David Root, Addgene plasmid 41393).

For virus production, Human embryonic kidney 293T (HEK293T) cells were cultured in DMEM supplemented with 10% FCS, 2 mM L-glutamine, 100 U/ml penicillin, 100 mg/mL streptomycin and 0.11 mg/mL sodium pyruvate and were seeded to achieve 70–80% confluence at time of transfection. HEK293T cells were transfected using calcium phosphate co-precipitation of the five plasmids (pL40c/pCW57.1 with TAT, REV, GAG/POL and VSV-G) at a mass ratio of 24 μg:1.2 μg:1.2 μg:1.2 μg:2.4 mg per 150 mm–diameter plate of cells. Viral supernatant was harvested after 24 h and subsequently every 12 h for 36 h before concentration with Centricon Plus-70 100-kDa filter (Millipore), using the manufacturer’s instructions. Concentrated viral particles were stored at 70°C before lentiviral transduction. Cell lines were transduced with concentrated virus in the presence of 8 μg/mL polybrene and 1x Stemspan CD34^+^ expansion supplement (StemCell Technologies) by spinoculation at 1,500g for 50 min on RetroNectin (Takara) coated plates. After 12–16 h incubation at 37°C, viral medium was exchanged for fresh medium. Cells were cultured for 3 days prior to treatment with 1.5 μg/mL doxycycline (Merck), with a further treatment after an additional 48 h. After 3 days doxycycline treatment FACS was performed to isolate GFP+ (pCW57.1 dnFOS & dnCEBP). For colony formation assays – sorted cells were seeded at 5000 cells/ml in Methocult Express (StemCell Technologies) with 1.5 μg/mL doxycycline and counted after 12 days.

#### Mini-shRNA screen in primary cells

For shRNA mini screen in primary cells, cells were transduced with a library of a mixed shRNA pool and were cultured for 15 days with or without 1.5 μg/mL doxycycline added every 3 days. Doxycycline induced cells were then sorted for venus+/tomato+ cells and DNA was extracted from these sorted cells and the uninduced population using a DNeasy Blood and Tissue kit (Qiagen).

#### ATAC-seq analysis of primary cells

Omni ATAC-seq was performed as in Corces et al.^56^ Briefly, cells were washed in ATAC resuspension buffer (RSB) (10mM Tris-HCl pH7.5, 10mM NaCl and 3mM MgCl_2_) and then lysed for 3 min on ice in RSB buffer with 0.1% NP-40, 0.1% Tween 20. Then the cells were washed with 1mL of ATAC wash buffer consisting of RSB with 0.1% Tween 20. Then the nuclear pellet was resuspended in ATAC transposition buffer consisting of 25μL TD buffer and a concentration of Tn5 transposase enzyme (Illumina) related to the number of input cells, 16.5 μL PBS, 5 μL water, 0.1% tween 20 and 0.01% digitonin and then incubated on a thermomixer at 37°C for 30 min. The transposed DNA was then amplified by PCR amplification which was assessed by a qPCR side reaction. The library was purified using a QIAquick PCR cleanup kit (QIAGEN) followed by ampure (Beckman Coulter) and analyzed on a Next Seq 2000 75 using a NextSeq 500/550 High output kit.

#### RNA-seq of primary cells

Two different methods were used to prepare RNA. RNA was extracted from primary cells using a RNeasy Micro Plus kit (QIAGEN) where less than 50,000 cells were harvested, and a RNeasy Micro kit (QIAGEN) was used for larger cell numbers. After quantification by nanodrop and QC using an Agilent RNA 6000 Pico Kit (Agilent, bioanalyser), libraries for next generation sequencing were prepared using the NEBnext Ultra II Directional RNA Library Prep Kit for Illumina (NEB) with the NEBNext rRNA Depletion Kit v2 for low RNA input (<100 ng RNA), or the Total RNA Ribo-zero library preparation kit (with ribosomal RNA depletion) (Illumina) for higher RNA input. Libraries were quantified using the High Sensitivity DNA kit (Agilent) and Kapa Library Quantification kit (Roche) prior to paired end sequencing on a Next Seq 2000 (PE 75) with a NextSeq High 150 v2.5 kit.

#### Proximity ligation assay (PLA) of CBFb:RUNX1 interaction

1.5 3 10^5^ cells were adhered to microscope slides using a Cytospin cytocentrifuge (Thermo Fisher Scientific) for 3 min at 800 xG and fixed in 4% formaldehyde (Pierce) for 15 min. Cells were permeabilised in 0.1% Triton X-100 and nonspecific staining was prevented by incubation in 3% bovine serum albumin. Anti-CBFβ (sc-56751; Santa Cruz Biotechnology) at 1:100 and anti-RUNX1 (ab23980, Abcam) at 1:100 primary antibodies were applied for 1 h at room temperature in PLA antibody diluent solution. Probes, ligation, and amplification solutions (Duolink; Sigma-Aldrich) were then applied at 37°C according to the manufacturer’s instructions, and the slides were mounted in Duolink mounting medium with DAPI (Sigma-Aldrich). Slides were visualised using a Zeiss LSM 780 equipped with a Quasar spectral (GaAsP) detection system, using a Plan Achromat 40 31.2 NA water immersion objective, Lasos 30 mW Diode 405 nm, Lasos 25 mW LGN30001 Argon 488, and Lasos 2 mW HeNe 594 nm laser lines. Images were acquired using Zen black version 2.1. Post-acquisition brightness and contrast adjustment was performed uniformly across the entire image.

#### RUNX1 ChIP seq from ITD-14 patient cells

Primary AML cells were cultured on hMSC feeders as described above in the following media: SFEMII (StemCell Technologies), 1 μM UM729 (StemCell Technologies), 750 nM StemReginin 1 (StemCell Technologies) supplemented with 150 ng/mL SCF, 100 ng/mL TPO, 10 ng/mL IL-3, 10 ng/mL G-CSF (Perpro tech). After 1 passage (1 week) in culture cells were treated for 24 h with 10 μM CBFβi or 0.1% DMSO in the absence of UM729 and StemReginin 1. 2 million cells were harvested and crosslinked in 1% formaldehyde solution (methanol-free from Pierce, Thermo Scientific). 400 mM of glycine (Merck) was added, and cells were washed twice with PBS (Merck) after which pellets were frozen at −80°C.

Crosslinked cells were resuspended at 1x10^7^ cells/ml in Buffer A (10 mM HEPES, 10 mM EDTA, 0.5 mM EGTA, 0.25% Triton X-100, 1x complete mini protease inhibitor cocktail (PIC) (Merck) pH 8.0) and incubated at 4°C for 10 min prior to centrifugation at 500 xG for 10 min. This step was repeated with Buffer B (10 mM HEPES, 200 mM NaCl, 1 mM EDTA, 0.5 mM MEGTA, 0.01% Triton X-100, 1x PIC, pH 8.0) and after centrifugation 2 x 10^6^ cells were resuspended in 300 μL IP buffer I (25 mM Tris-HCl, 150 mM NaCl, 2 mM EDTA, 1% Triton X-100, 0.25% SDS, 1x PIC, pH 8.0) and sonicated using a Diagenode Bioruptor Pico sonicator for 4 cycles (30 s on 30 s off) before centrifugation for 10 min at 16,000 xG. The supernatant was then collected and 600 μL IP Buffer II (25 mM Tris-HCl, 150 mM NaCl, 2 mM EDTA, 1% Triton X-100, 7.5% Glycerol, 1x PIC, pH 8.0) was added prior to immunoprecipitation.

For immunoprecipitations 15 μL of Dynabeads-Protein G were washed twice with 500 μL 50 mM citrate phosphate buffer pH 5 and resupended in 15 μL citrate phosphate buffer with 4 μg anti-RUNX1 antibody (ab23980, Abcam) and 0.5% acetyl-BSA before incubation at 4°C for 2 h. After incubation, dynabeads were washed with 500 μL pH 5 citrate phosphate buffer and resuspended in 15 μL citrate phosphate buffer with 0.5% BSA before 555 μL of sonicated chromatin was added and incubated at 4°C for ~16 h.

After the incubation the dynabeads are washed sequentially with 500 μL of: Wash buffer 1 (20 mM Tris-HCl, 150 mM NaCl, 2 mM EDTA, 1% Triton X-100, 0.1% SDS, pH 8.0) once, Wash buffer 2 (20 mM Tris-HCl, 500 mM NaCl, 20 mM EDTA, 1% Triton X-100, 0.1% SDS, pH 8) twice, LiCl buffer (10 mM Tris-HCl,250 mM LiCl, 1 mM EDTA, 0.5% NP-40, 0.5% Na-deoxycholate, pH 8.0) once, TE/NaCl wash buffer (10 mM Tris-HCl, 50 mM NaCl, 1 mM EDTA, pH 8.0) twice. After these washes DNA was eluted from the dynabeads using 100 mL elution buffer (100 μM NaHCO_3_, 1% SDS). 200 mM NaCl and 500 μg/mL proteinase K were added to the eluant and the sample was reverse crosslinked at 65°C for >4h. DNA was then purified by ampure (1.8x).

Libraries for next generation sequencing were prepared using a Kapa Hyper prep kit (Roche) according to manufacturer’s instructions. 16 cycles of PCR amplification were used and 200–450 bp fragments were size selected by gel electrophoresis. Libraries were validated by qPCR and quantified using the High Sensitivity DNA kit (Agilent) and Kapa Library Quantification kit (Roche) prior to sequencing on a Nextseq 2000 75 using a NextSeq 500/550 High output kit.

#### Single cell treatment scRNA-Seq analysis of CBFβi treated FLT3-ITD+ AML

Primary AML cells for scRNA-seq were cultured on hMSC feeders as described above in the following media: SFEMII (StemCell Technologies), 1 μM UM729 (StemCell Technologies), 750 nM StemReginin 1 (StemCell Technologies) supplemented with 150 ng/mL SCF, 100 ng/mL TPO, 10 ng/mL IL-3, 10 ng/mL G-CSF (Perpro tech). After 1 passage (1 week) in culture cells were treated for 24 h with 10 μM CBFβi or 0.1% DMSO in the absence of UM729 and StemReginin 1. After treatment cells were sorted for CD45 using magnetic beads (Miltenyi Biotec). Cells were loaded on a Chromium Single Cell Instrument (10X Genomics), to recover 5000 single cells. Library generation was performed using the Chromium single cell 3′ library and gel bead kit v3.1. Illumina sequencing was performed on a NovaSeq 6000 S1 run in paired-end mode for 150 cycles at a depth of 20000 reads per cell.

#### Bulk RNA-Seq data analysis

Raw paired-end reads were trimmed to remove low-quality sequences and adaptors using Trimmomatic v0.39.^39^ Reads were then aligned to the human genome (version hg38) using HISAT2 v2.2.1^40^ with default settings. Counts were generated with featureCounts v2.0.1^41^ using gene models from ensembl as the reference transcriptome. Differential gene expression analysis was carried out using Limma-Voom v3.50.3^42^ in R v4.1.2.

#### Single-cell RNA-Seq analysis

Fastq files from single-cell sequencing experiments were aligned to the human genome (version hg38) using the count function in CellRanger v5.0.1 from 10x genomics^43^ using gene models from ensembl as the reference transcriptome. Analysis was then carried out using the Seurat package v4.3.0^44^ in R v4.1.2. Cells from CBFbi treated and untreated samples were filtered to remove cells with less than 500 and more than 6000 detected genes, as well as cells with more than 20% of reads aligned to mitochondrial transcripts. The filtered cells were then combined into a single dataset for downstream analysis. UMI counts were normalized using the NormalizeData function with default settings. The cell cycle stage for each cell was inferred using the CellCycleScoring function in Seurat. This score was then used to remove the possible effect of cell cycle stage on the analystrimmomis by linear regression using the ScaleData function. Clustering was then carried out using the FindNeighbors and FindClusters commands, using the top 20 principal components and a cluster resolution value of 0.25. Differential gene expression analysis was carried out for each single cell cluster, comparing CBFBi treated cells to untreated cells using the FindMarkers command. A gene with a log2 fold-change of at least 0.25 and an adjusted p value less than 0.1 were considered to be differentially expressed. Kyoto Encyclopedia of Genes and Genomes (KEGG) pathway analysis was then carried out on the sets of differentially expressed genes using the ClueGO package v2.5.0^45^ in Cytoscape v3.9.1.^57^ Cell trajectory (pseudotime) analysis was carried out using Monocle3 v1.3.1.^46^ To do this, the processed data from Seurat was first divided into two objects corresponding to CBFBi treated and untreated samples. These were then imported into Monocle using the as.cell_data_set function in SeuratWrappers. Single cell trajectories were calculated using the cluster_cells and learn_graph commands in Monocle. Pseudotime was then calculated by rooting the trajectory at the earliest point of the inferred trajectory that occurred in the early progenitor cells.

#### shRNA data analysis

To calculate read counts from shRNA experiments, 75bp single-end reads in fastq format were first processed to remove the first and last 25bp from each sequence, corresponding to the regions flanking the shRNA sequence that are common across all reads. The shRNA sequences were then compared to the library of oligonucleotide sequences used in the experiment, allowing for only a single base mismatch. Read counts were normalized using upper-quartile normalization using the edgeR package v3.36.0^47^ in R v4.1.2. To calculate fold-changes between doxycycline induced and non-induced cells, the normalized counts were fitted to a generalized linear model using edgeR. A shRNA sequence was deemed to have been lost if it had a log2 fold-change less than −1 between induced and non-induced samples.

#### ATAC-seq data analysis

Single-end reads from ATAC-Seq experiments were processed to remove low-quality sequences and Nextera ATAC adaptors using Trimmomatic. Reads were then aligned to the human genome (version hg38) using Bowtie2 v2.2.5^48^ with the option --very-sensitive-local. Potential PCR duplicates were identified and removed from alignments using Picard MarkDuplicates v2.26.10 (http://broadinstitute.github.io/picard). Peaks were called using MACS2 v2.2.7.1^49^ with the parameters --nomodel -B --trackline. The resulting peaks were then filtered to remove any peak with a peak height less than 10 or were found in the hg38 blacklist^58^ Where replicates were available, only peaks that passed these filters in both replicates were retained. A peak union was then created for each set of experiments by first extending the peak region by 200bp either side of the peak summit. Overlapping peaks were then combined using the merge function in bedtools v2.30.0.^50^ The distance between the peak summit and the closest gene was then calculated using the annotatePeaks.pl function in Homer v4.9.1.^51^ A peak was classified as distal if it was at least 1.5kb from the nearest transcriptional start site (TSS), and as promoter-proximal otherwise. Distal and promoter-proximal peaks were treated separately in downstream analyses.

Differential peak analysis was carried out by first counting the number of reads aligned to each peak using featureCounts. These were then normalized as counts-per-million using the edgeR package in R. In cases where replicates were available, fold-differences and statistical values were calculated using Limma-Voom. Where only single experiments were available, a simple fold-difference was calculated by subtracting the log2-normalized count from the treatment sample from the control (NTC, empty-vector as appropriate). Read density plots were created by first ranking peaks according to fold-difference. The read counts were then retrieved in a 2kb window centered on the peak summit using the annotatePeaks.pl function in Homer with the options -size 2000 -hist 10 -ghist -bedGraph with the bedGraph files produced by MACS2 as input. These were then plotted as a heatmap using Java TreeView v1.1.6r4.^52^ Motif enrichment analysis was carried out in the set of gained and lost peaks using the findMotifsGenome.pl function in Homer with the options -size 200 -noknown.

#### ChIP-seq data analysis

Reads were trimmed to remove low-quality sequences using Trimmomatic and aligned to the human genome (version hg38) using Bowtie2 with the --very-sensitive-local paramter. Potential PCR duplicates were identified and removed from alignments using Picard MarkDuplicates. Peaks were then called using MACS2 with the settings -B --trackline. Read density plots were created by first ranking peaks by tag count, and then retrieving the read density in a 2kb region centered on the peak summit using the annotate-Peaks.pl function in Homer. These were then shown as a heatmap in Java TreeView.

#### Re-analysis of public DNaseI-Seq data

DNaseI-Seq data from FLT3-ITD, FLT3-ITD + NPM1 and healthy PBSCs from Assi et al. (2019) were downloaded from GEO using the accession number GSE108316. Reads were then trimmed using Trimmomatic and aligned to the human genome using Bowtie2 using the parameter --very-sensitive-local. Peaks were called using MACS2 with the options --keep-dup all --nomodel -q 0.0005 --call-summits -B --trackline. In order to ensure that peak coordinates were accurate and representative of all patients, alignments were merged to create a single dataset. Peak calling was then repeated on this dataset and the resulting peak coordinates were used as the reference peak positions for all further analysis. The peaks from each sample were filtered to remove peaks in the hg38 blacklist, and only peaks that were found in at least 50% of all patients from their respective groups (ITD, ITD-NPM1 or PBSC) were retained for further analysis. Differential peak analysis was carried out by first classifying peaks as either distal, or as promoter-proximal as described for the ATAC-Seq data above and processed separately. The average read count in a 400bp window centered on the peak summit was then retrieved using the annotatePeaks.pl function in Homer with the options -size 400 -bedGraph and using the bedGraph files produced by MACS2. These counts were then normalized to the average read count across samples. The average read count for each group (ITD/ITD-NPM1 or PBSC) was then calculated, and further log2-transformed as log2(average read count +1). A peak was deemed to be specific to a group if it had a fold-difference greater than 3 in either the ITD or ITD-NPM1 groups compared to healthy PBSCs. DNaseI footprinting was carried out on the merged alignments from ITD, ITD-NPM1 and PBSCs using the wellington algorithm v0.2.0^11^ using the options -fdrlimit −5 -fp 11,32,2. The resulting set of footprints were then combined using the bedtools merge command.

#### Construction of gene regulatory networks

Gene Regulatory Networks (GRNs) were made using custom Python scripts that have been made publicly available (see code and data availability section). To construct ITD/ITD-NPM1 and PBSC specific networks, we first identified sets of DNaseI Hypersensitive Sites (DHSs) from Assi et al.^2^ that had a fold-difference greater than 3 between cell types. The genomic positions of transcription factor binding motifs were then retrieved from within these sites using the annotatePeaks.pl function in Homer and exported as BED files using the -mbed option. To ensure that the motif sequences used in each of these networks were constant, we used the set of probability weight matrices that were defined in Assi et al.^2^ and have been made available for further use (see code and data availability section). These motif positions were then further refined by only keeping those that were found within DNaseI footprints. To ensure that DHSs were assigned to the correct gene, we used processed promoter-capture HiC data from patients with FLT3-ITD and healthy CD34 positive cells that were analyzed by Assi et al.^2^ In cases where no HiC annotation was present for a given DHS, the peak was instead assigned to the closest gene. To ensure that only genes that were actually expressed in our data were included in the GRN, we used RNA-Seq data from Assi et al.^2^ which includes data from the same set of patients that were used to construct the GRN. These were processed as described above, and only genes that were expressed with a Fragments Per Kilobase per Million mapped reads (FPKM) value greater than 1 in either patients with ITD/ITD-NPM1 or healthy PBSCs were included. A network was then constructed for each set of specific and shared DHSs where transcription factor genes are represented by nodes, and the presence of a footprinted binding motif in a DHS targeting a gene is represented by a directed edge. Members of the same transcription factor families are known to bind to highly similar or identical motifs^59^ making the definitive identification of specific transcription factor genes from motif data alone difficult. To account for this in our GRNs, we grouped members of the same family into groups with one binding motif representing the entire set of transcription factor genes. The most highly expressed member of that family was used as the source node. The GRN graph was then exported as a JSON file and visualised using Cytoscape.

#### Transcription factor module similarity

To measure if the sets of genes that were targeted by different transcription factor families were similar, we first extracted the module for each TF family. A module here is defined as the complete set of target genes from the GRN and includes both transcription factor and non-transcription factor genes (Figure 3A). The overlap of these modules was then measured using the Jaccard similarity index using the formula

$$Jaccard\;Index=\frac{\left|A\cap B\right|}{\left|A\cup B\right|}$$

Where A and B are the sets of genes for two different transcription factor modules. This was calculated for each pair of transcription factor modules, resulting in a matrix of Jaccard Index values. These were then hierarchically clustered using complete linkage of the Euclidean distance in R and shown as a heatmap (Figures 3B–3E).

### QUANTIFICATION AND STATISTICAL ANALYSIS

Statistical analysis methods used in bioinformatic analysis are listed in the bioinformatic methods. For other figures, data are expressed as mean ± S.D. unless otherwise noted. Exact numbers of biological and technical replicates for each experiment are reported in the figure legends. p values less than 0.05 were considered statistically significant by unpaired, two-tailed Student’s t test and are indicated in the relevant figures.

### ADDITIONAL RESOURCES

None.

## Supplementary Material

1

2

3

4

5

6

7

8

## Figures and Tables

**Table:** KEY RESOURCES TABLE

REAGENT or RESOURCE	SOURCE	IDENTIFIER
Antibodies		
EGR1 Antibody (588)	Santa Cruz Biotechnology	Cat# sc-110; RRID:AB_2097174
E4BP4 Polyclonal Antibody (NFIL3)	Bethyl Laboratories	Cat# A302-606A; RRID:AB_10555364
AML1 Antibody	Cell Signaling Technology	Cat# 4334; RRID:AB_2184099
Anti-GAPDH Antibody [6C5]	Abcam	Cat# ab8245; RRID:AB_2107448
PEBPβ2 Antibody (141,4,1)	Santa Cruz Biotechnology	Cat# sc-56751; RRID:AB_781871
Anti-RUNX1/AML antibody	Abcam	Cat# ab23980; RRID:AB_2184205
Anti-rabbit IgG HRP-linked antibody	Cell Signaling Technology	Cat# 7074; RRID:AB_2099233
Anti-mouse IgG HRP-linked antibody	Cell Signaling Technology	Cat# 7076; RRID:AB_330924
Anti-goat IgG HRP-linked antibody	Jackson ImmunoResearch	Cat# 115-035-062; RRID:AB_2338504
Bacterial and virus strains		
XL-gold bacteria	Agilent	200315
Chemicals, peptides, and recombinant proteins		
Recombinant human TPO	PeproTech	300–18
Recombinant human IL-3	PeproTech	200–03
Recombinant human G-CSF	PeproTech	300–23
Recombinant human SCF	Peprotech	300–07
Recombinant human GM-CSF	PeproTech	300–03
FBS Qualified	Gibco	10270–106
HEPES solution 1M pH7.4	Sigma	H0887
Pen Strep	Gibco	15070–063
MACS BSA Stock Solution	Miltenyi Biotech	130-091-376
RPMI 1640 Medium	Sigma Aldrich	R8758
Dulbeccos Modified Eagles Medium	Sigma Aldrich	D6546
Iscoves MDM	Merck	I7633
StemSpan SFEM II	StemCell Technologies	09605
UM729	StemCell Technologies	72332
StemRegenin 1	StemCell Technologies	72344
StemSpan CD34^+^ Expansion Supplement (10x)	StemCell Technologies	02691
L-Glutamine	Gibco	25030081
BsmBI	Thermo	ER0451
T4 DNA Ligase kit	Thermo	EL0011
Calcium Chloride dihydrate	Sigma Aldrich	C3306
Sodium Chloride	Acros Organics	207790050
HEPES	Sigma Aldrich	H3375
Sodium phosphate dibasic	Sigma Aldrich	S3397
Polybrene	Sigma Aldrich	TR-1003-G
RetroNectin Recombinant Human Fibronectin Fragment	Takara	T100A
Doxycycline	Sigma Aldrich	D5207
Phosphate Buffered Saline	Merck	806552
Ex Taq DNA polymerase	Takara	RR001A
Nusieve 3:1 Agarose	Lonza	50090
Ampure XP SPRI Reagent	Beckman Coulter	A63881
Methocult H4100	StemCell Technologies	H4100
Methocult Express	StemCell Technologies	04437
Laemmli buffer	Bio-Rad	1610747
Enhanced chemiluminescent reagent	Cytiva	RPN2134
Tris-HCl	Fisher Bioreagents	BP153-1
Tween 20	Sigma Aldrich	P2287
Restore Stripping Buffer	Thermo Scientific	21059
β-mercaptoethanol	Sigma Aldrich	M3148
Hydrocortisone	Sigma Aldrich	H0888
BCI	Selleckchem	S2837
Quizartinib	Selleckchem	S1526
AI-4-88	Illendula et al.^27^	N/A
AI-14-91	Illendula et al.^27^	N/A
DMSO	Merck	D2650
Trypan Blue	Merck	T8154
Magnesium chloride	Fisher Scientific	M/0600/53
Tn5 transposase enzyme and TD buffer	Illumina	15027865/6
Nonidet P-40	BDH Laboratory Supplies	56009
Digitonin	Promega	G944A
NEBNext^®^ High-Fidelity 2X PCR Master Mix	New England Biolabs	M0541S
16% formaldehyde (methanol free)	Thermo Scientific	28906
Triton X-100	Sigma Aldrich	T8787
Glycine	Merck	357002
EDTA	Sigma Aldrich	E5134
EGTA	Sigma Aldrich	E3889
Complete Mini Protease Inhibitor Cocktail	Merck	04693124001
Sodium dodecyl sulfate	Sigma Aldrich	L5750
Glycerol	Fisher Scinetific	G/0650/17
Dynabeads-Protein G	Invitrogen	10004D
Albumin, Acetylated from bovine serum	Merck	B2518
Phosphate citrate buffer tablet	Sigma Aldrich	P4809
Lithium chloride	Sigma Aldrich	L9650
Sodium deoxycholate	Alfa Aesar	B20759
Sodium bicarbonate	Sigma Aldrich	S6297
Critical commercial assays		
QIAquick Gel Extraction kit	Qiagen	28706
EndoFree Plasmid Maxi Kit	Qiagen	12362
DNeasy Blood and Tissue Kit	Qiagen	69504
NextSeq 500/550 High output v2.5 kit (75 cycles)	Illumina	20024906
NextSeq 500/550 High output v2.5 kit (150 cycles)	Illumina	20024907
Turbo transfer packs	Bio-rad	1704156
Mini PROTEAN TGX Gels	Bio-rad	4561096
QIAquick PCR clean up kit	Qiagen	28006
RNeasy Micro Plus kit	Qiagen	74034
RNeasy Micro kit	Qiagen	74004
NEBnext Ultra II Directional RNA Library Prep Kit for Illumina	New England Biolabs	E7760
NEBNext^®^ rRNA Depletion Kit v2 for	New England Biolabs	E7400
TruSeq RNA Library Preparation Ki	Illumina	RS-122-2001
Duolink *in situ* detection reagents Red	Sigma Aldrich	DUO92008
Duolink DAPI	Sigma Aldrich	DUO82040
Duolink *in situ* PLA probe anti-mouse MINUS	Sigma Aldrich	DUO92004
Duolink *in situ* PLA probe anti-rabbit PLUS	Sigma Aldrich	DUO92002
Kapa Hyper prep kit	Roche	07962363001
High Sensitivity DNA kit	Agilent	5067-4626
Kapa Library Quantification kit	Roche	07960204001
Chromium single cell 3ʹ library and gel bead kit v3.1	10x Genomics	PN-1000128
Ampure XP	Beckman Coulter	A63881
Deposited data		
ATAC and RNA-seq data	This paper	GEO database: GSE2367751
Experimental models: Cell lines		
MOLM14	DMSZ	ACC 777
MV4-11	DMSZ	ACC 102
HEK293T	DMSZ	ACC 305
Kasumi-1	DMSZ	ACC 220
SKNO-1	DMSZ	ACC 690
KG1a	DMSZ	ACC 421
P31/FUJ	JCRB Cell Bank	JCRB0091
Experimental models: Organisms/strains		
Mouse NSG (NOD.Cg-Prkdcscid Il2rg tm1Wjl/SzJ)	In house breeding – Newcastle University	N/A
Mouse (Rag2−/−Il2rg −/− 1293Balb/c)	In house breeding – Newcastle University	N/A
Oligonucleotides		
shRNA oligonucleotides	https://felixfadams.shinyapps.io/miRN/	Data S2
Software and algorithms		
Prism	Graphpad	v9.4.1
GRN construction scripts	https://doi.org/10.5072/zenodo.268	https://github.com/petebio/Gene_regulatory_network_analysis
Trimmomatic	Bolger et al.^39^	v0.39
HISAT2	Kim et al.^40^	v2.2.1
featureCounts	Liao et al.^41^	v2.0.1
Limma-Voom	Law et al.^42^	v3.50.3
R	https://www.r-project.org/	v4.1.2
CellRanger	Zheng et al.^43^	v5.0.1
Seurat package	Hao et al.^44^	v4.3.0
ClueGO	Bindea et al.^45^	v2.5.0
Monocle3	Qui et al.^46^	v1.3.1
edgeR	Robinson et al.^47^	v3.36.0
Bowtie2	Langmead and Salzberg^48^	v2.2.5
Picard MarkDuplicates	http://broadinstitute.github.io/picard	v2.26.10
MACS2	Zhang et al.^49^	v2.2.7.1
bedtools	Quinlan and Hall^50^	v2.30.0
Homer	Heinz et al.^51^	v4.9.1
Java TreeView	Saldanha^52^	v1.1.6r4
Wellington Algorithm	Piper et al.^11^	v0.2.0
Cytoscape	https://cytoscape.org/	v3.10.0

## Highlights

- A shRNAi drop-out screen for FLT3-ITD AML based on gene regulatory networks
- AML-specific GRN analysis predicts regulatory modules required for leukemic growth
- Perturbation of key transcription factor modules highlights module connectivity
- RUNX1 stabilizes the GRN of FLT3-ITD AML, and its removal leads to cell death
