# Supplementary material for: Gene regulatory network analysis predicts cooperating transcription factor regulons required for FLT3-ITD+ AML growth
Source: Cell Rep. Author manuscript; Available in PMC 2024 Feb 18. (PMC10874628; doi:10.1016/j.celrep.2023.113568)
Supplement: 1 [file NIHMS1954801-supplement-1.pdf]

Cell Reports, Volume *42*

Supplemental information

Gene regulatory network analysis predicts cooperating transcription factor regulons required for FLT3-ITD+ AML growth

Daniel J.L. Coleman, Peter Keane, Rosario Luque-Martin, Paulynn S. Chin, Helen Blair, Luke Ames, Sophie G. Kellaway, James Griffin, Elizabeth Holmes, Sandeep Potluri, Salam A. Assi, John Bushweller, Olaf Heidenreich, Peter N. Cockerill, and Constanze Bonifer

Supplementary Materials

List of Supplementary Materials

1. Supplementary Table 1: Patient Data

2. Supplementary Figures and Figure legends

3. Supplementary Reference

Supplementary Table S1:

Patient Data for primary cell samples used in this study (related to all figures)

Patient Code	Mutations	Sex	wbc	Case
ITD-12	46XX, 7q-, i(13), DNMT3A, U2AF1 and FLT3-ITD	F	65	Presentation
ITD-13	46XY, FLT3-ITD, JAK2, CEBPA,	M	376	Presentation
ITD-14	46XX, FLT3-ITD, NPM1, ANKRD26, CEBPA (10%), ESXH2, TET2x2, ZRSR2	F	300	Presentation
ITD-15	46 XY, FLT3 ITD, CHEK2, CUX1	M	17	Relapse
ITD-NPM1-2	46XX, FLT3-ITD, NPM1	F	7	Relapse
ITD-NPM1-6	46XX, FLT3-ITD, NPM1, WT1, DNMT3A, TET2, PHF6	F	195	Presentation
RUNX1(x2)-1	46XY del 21q, RUNX1x2, TET2, STAG2, BCOR, IKZF1, EZH2	M	78	Presentation after MDS

Information on patient samples used in this paper. For other patients used in analysis see Assi et al 2019[1].

Figure S1

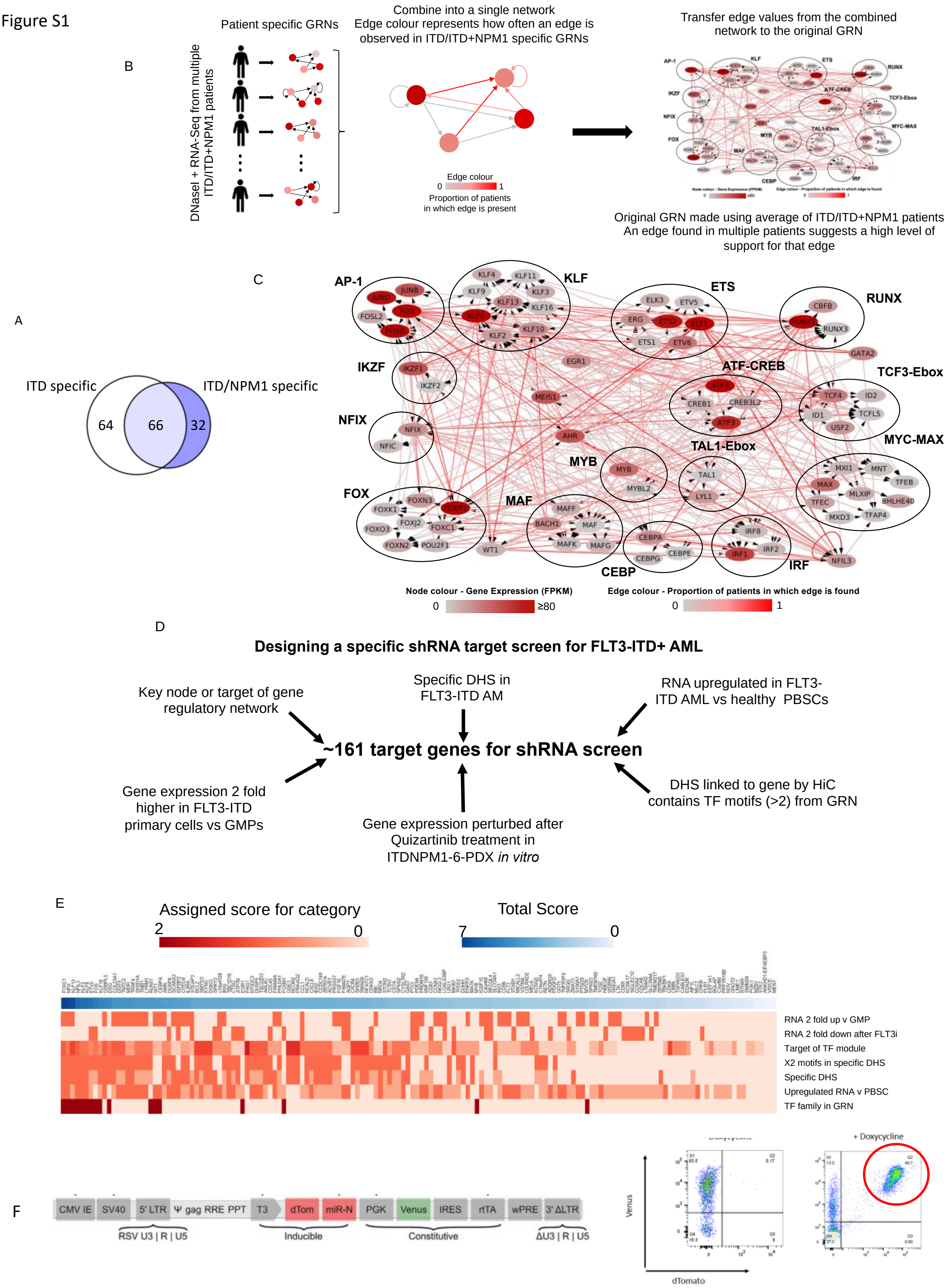

Figure S1 (related to Figures 1&2). Design of shRNA screen targeting the FLT3-ITD GRN

A: Overlap of shared edges in FLT3-ITD and FLT3-ITD/NPM1 AML-specific TF networks. B: Scheme of GRN comparison between nine patients. C: Combined gene regulatory network showing all edges that are found in any ITD/ITD-NPM1 specific GRN. The color of the edges

represents the proportion of patients in which an edge is found. A high value indicates that an edge is found in multiple AML patients, suggesting a high level of support for that edge. D: Filtering strategy for target gene identification. Target genes for the shRNA screen were scored based on multiomic data from Assi et al 2019[1] and genes perturbed after treatment of ITD-NPM1-6-PDX primary cells treated with 10 nM quizartinib in vitro. E: Heatmap of scoring of genes selected for the FLT3-ITD screen based upon the criteria shown in D. Red heatmap shows the score of the gene for each category with the blue bar showing the total score. F: Structure of shRNA expressing lentivirus (pL40c) and representative flow cytometry images of marker gene expression after library transduction with and without Doxycycline treatment.

Figure S2

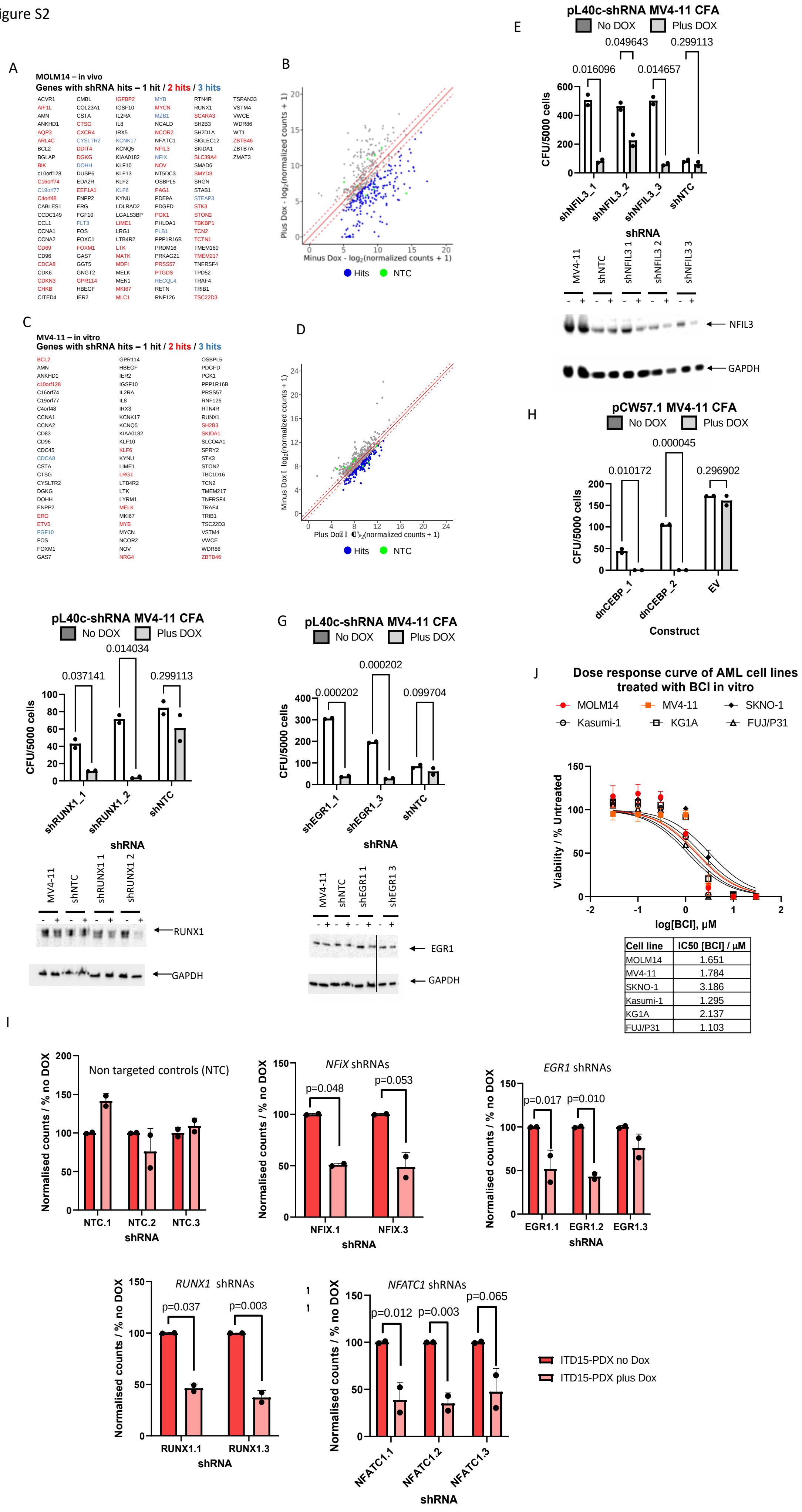
A
MOLM14 – in vivo
Genes with shRNA hits – 1 hit / 2 hits / 3 hits
B
Plus Dox - log2(normalized counts + 1)
Minus Dox - log2(normalized counts + 1)
Hits
NTC
C
MV4-11 – in vitro
Genes with shRNA hits – 1 hit / 2 hits / 3 hits
D
Hits
NTC
E
pL40c-shRNA MV4-11 CFA
No DOX
Plus DOX
0.016096
0.049643
0.014657
0.299113
CFU/5000 cells
shNFIL3_1
shNFIL3_2
shNFIL3_3
shNTC
shRNA
MV4-11
shNTC
shNFIL3 1
shNFIL3 2
shNFIL3 3
NFIL3
GAPDH
H
pCW57.1 MV4-11 CFA
No DOX
Plus DOX
0.010172
0.000045
0.296902
CFU/5000 cells
dnCEBP_1
dnCEBP_2
EV
Construct
F
pL40c-shRNA MV4-11 CFA
No DOX
Plus DOX
0.037141
0.014034
0.299113
CFU/5000 cells
shRUNX1_1
shRUNX1_2
shNTC
shRNA
MV4-11
shNTC
shRUNX1 1
shRUNX1 2
RUNX1
GAPDH
G
pL40c-shRNA MV4-11 CFA
No DOX
Plus DOX
0.000202
0.000202
0.099704
CFU/5000 cells
shEGR1_1
shEGR1_3
shNTC
shRNA
MV4-11
shNTC
shEGR1 1
shEGR1 3
EGR1
GAPDH
J
Dose response curve of AML cell lines treated with BCI in vitro
MOLM14
MV4-11
SKNO-1
Kasumi-1
KG1A
FUJ/P31
Viability / % Untreated
log[BCI], µM
Cell line | IC50 [BCI] / µM
MOLM14 | 1.651
MV4-11 | 1.784
SKNO-1 | 3.186
Kasumi-1 | 1.295
KG1A | 2.137
FUJ/P31 | 1.103
I
Non targeted controls (NTC)
Normalised counts / % no DOX
NTC.1
NTC.2
NTC.3
shRNA
NFIX shRNAs
p=0.048
p=0.053
NFIX.1
NFIX.3
EGR1 shRNAs
p=0.017
p=0.010
EGR1.1
EGR1.2
EGR1.3
RUNX1 shRNAs
p=0.037
p=0.003
RUNX1.1
RUNX1.3
NFATC1 shRNAs
p=0.012
p=0.003
p=0.065
NFATC1.1
NFATC1.2
NFATC1.3
ITD15-PDX no Dox
ITD15-PDX plus Dox

Figure S2 (Related to Figure 2): Screening results and manual validation of selected hits in FLT3-ITD cell lines and primary cells

A: MOLM14 in vivo screen results showing genes with shRNAs lost in the population, genes with multiple shRNA hits are highlighted. B: Scatter plot showing log2 shRNA frequency in MOLM14 in vivo screen, with lost shRNAs highlighted. C: MV4-11 in vitro screen results showing genes with shRNAs lost in the population, genes with multiple shRNA hits are highlighted. D: Scatter plot showing log2 shRNA frequency in MV4-11 in vitro screen, with lost shRNAs highlighted. E-H validation of GRN targets in MV4-11 cell lines, performed in duplicate for each construct, p-values were calculated using Student's t-test. Constructs that did not show a decrease in protein expression after 72 h induction were excluded from the analysis. E: Colony formation assays of MV4-11 cells transduced with shRNA targeting NFIL3. Induction of the shRNA knockdown of NFIL3 by doxycycline caused a decrease in colony formation compared to uninduced cells. The Western blot below shows a decrease in NFIL3 protein expression after 72 h induction, with GAPDH included as a control. F: Colony formation assays of MV4-11 cells transduced with shRNA targeting RUNX1. Induction of the shRNA knockdown of RUNX1 by doxycycline caused a decrease in colony formation compared to uninduced cells. The Western blot below shows a decrease in RUNX1 protein expression after 72 h induction, with GAPDH included as a control. G: Colony formation assays of MV4-11 cells transduced with shRNA targeting EGR1. Induction of the shRNA knockdown of EGR1 by doxycycline caused a decrease in colony formation compared to uninduced cells. The Western blot below shows a decrease in EGR1 protein expression after 72 h induction, with GAPDH included as a control. Note that EGR1 and EGR3 shRNA images were spliced together (bar). H: Expression of a dominant negative CEBP in MV4-11 clones showed a decrease in colony forming ability after induction compared to uninduced cells and MV4-11s transduced with an empty vector control. I: Histograms of counts normalised to no dox control of shRNA mini-screen performed in primary FLT3-ITD PDX cells from ITD-15 with lentiviral vectors expressing DOX-inducible selected shRNAs as indicated. Experiments were performed in duplicate with several independent shRNAs. NTC: Non-targeted control. J: Dose response curves of cell lines treated with DUSP1/6 inhibitor BCI. FLT3-ITD cell lines show sensitivity to the inhibitor at a similar level to cell lines with MAPK activating mutations (Kasumi-1, P31/FUJ) whilst those without show marginally decreased sensitivity, although all AML cell lines respond to the inhibitor. Means calculated from n=3 are plotted with ± SEM and IC50 are shown below.

Figure S3

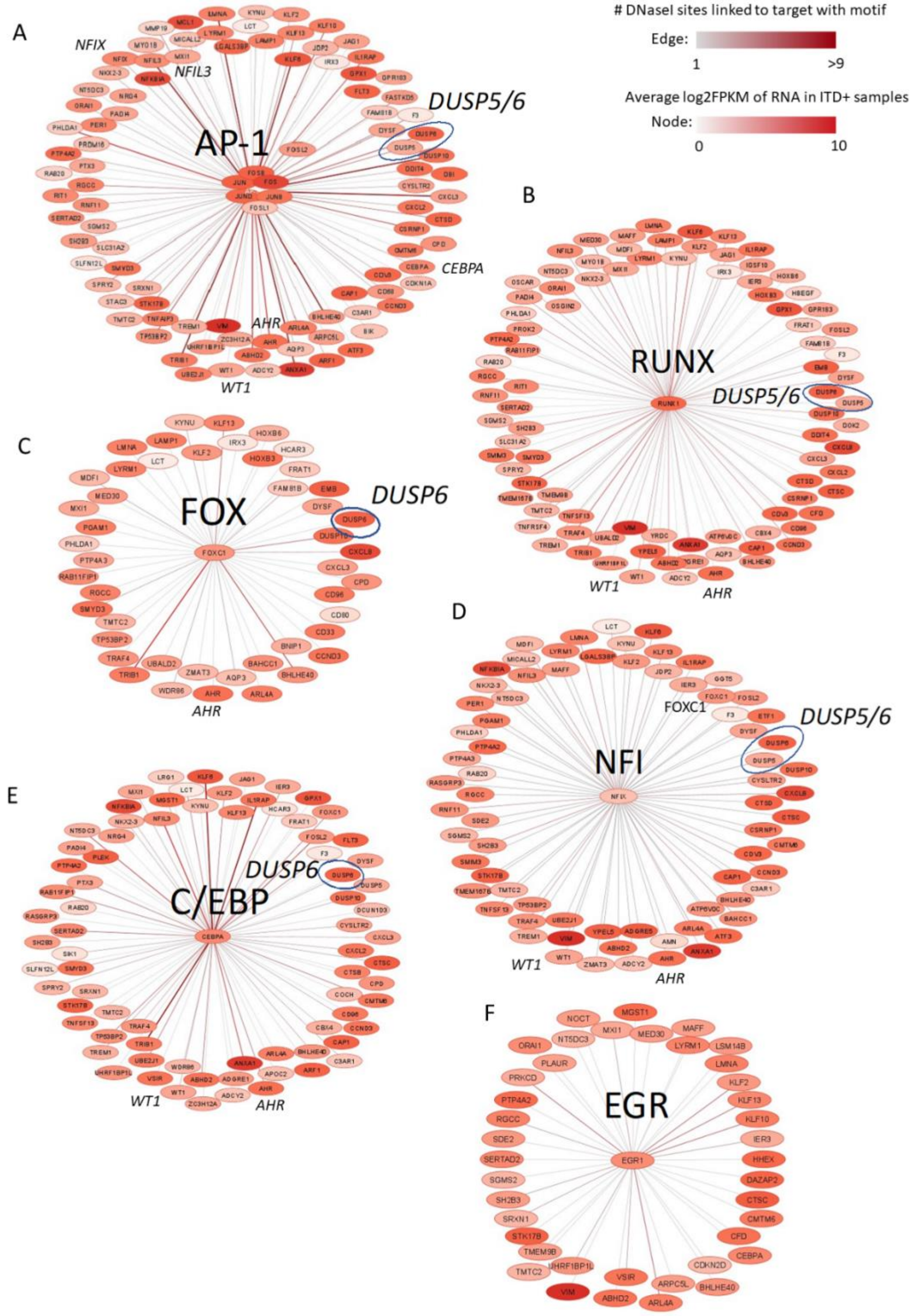

Figure S3 (related to figure 3) TF modules of upregulated genes in FLT3-ITD AML: A-F: AP-1, FOX, RUNX, NFI, C/EBP and EGR regulatory modules of FLT3-ITD AML specifically expressed genes as compared to PBSCs. Node colour indicates gene expression in FLT3-ITD+ AML samples (FPKM). Edges indicate an interaction between TF family and target genes, with the

colour of the edge denoting the number of regions of open chromatin containing the TF family motif linked to the target gene by HiC or nearest gene (within 200 kb). TFs that are associated with more than one module are highlighted.

Figure S4

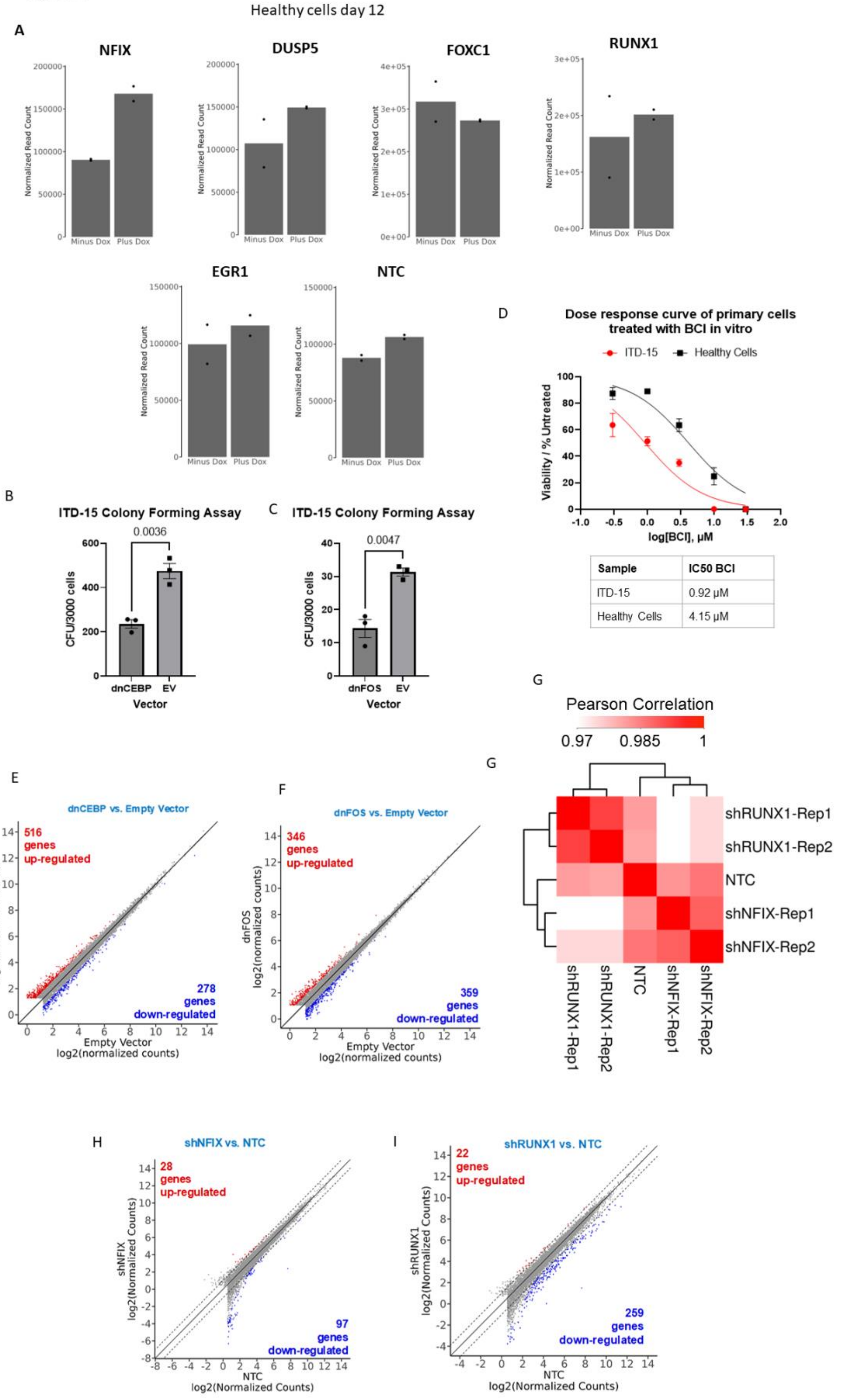
Healthy cells day 12
A
NFIX
DUSP5
FOXC1
RUNX1
EGR1
NTC
Normalized Read Count
Minus Dox
Plus Dox
B
ITD-15 Colony Forming Assay
0.0036
CFU/3000 cells
dnCEBP
EV
Vector
C
ITD-15 Colony Forming Assay
0.0047
CFU/3000 cells
dnFOS
EV
Vector
D
Dose response curve of primary cells treated with BCI in vitro
ITD-15
Healthy Cells
Viability / % Untreated
log[BCI], µM
Sample
IC50 BCI
ITD-15
0.92 µM
Healthy Cells
4.15 µM
E
dnCEBP vs. Empty Vector
516 genes up-regulated
278 genes down-regulated
dnCEBP log2(normalized counts)
Empty Vector log2(normalized counts)
F
dnFOS vs. Empty Vector
346 genes up-regulated
359 genes down-regulated
dnFOS log2(normalized counts)
Empty Vector log2(normalized counts)
G
Pearson Correlation
0.97
0.985
1
shRUNX1-Rep1
shRUNX1-Rep2
NTC
shNFIX-Rep1
shNFIX-Rep2
H
shNFIX vs. NTC
28 genes up-regulated
97 genes down-regulated
shNFIX log2(Normalized Counts)
NTC log2(Normalized Counts)
I
shRUNX1 vs. NTC
22 genes up-regulated
259 genes down-regulated
shRUNX1 log2(Normalized Counts)
NTC log2(Normalized Counts)

Figure S4 (related to Figure 4). TF perturbation experiments in primary cells

A: Bar charts showing the results of shRNA screen in healthy cells focused on 6 targets. DNA was harvested and libraries were prepared after 12 days of culture with or without doxycycline induction (n=2). B, C: Bar charts showing the effect of lentivirally transduced dominant negative CEBP (B) and FOS (C) peptide on colony forming ability of FLT3-ITD primary AML cells with and without Doxycycline stimulation. Experiments were performed in triplicate, mean colony formation per 5000 cells seeded are plotted and p values have been calculated by Student's t-test. Individual data points are plotted with the bar showing mean colony formation ± SEM. D: Dose response curve of treatment of FLT3-ITD primary AML cells and a healthy control with different concentrations of the DUSP inhibitor BCI as indicated. Means calculated from n=3 are plotted with ± SEM and IC50 are shown below. E,F: Scatter plot of mRNA expression patterns of FLT3-ITD primary AML cells with and without induction of dnCEBP and dnFOS compared to an empty vector control. H, I: Scatter plot of gene expression patterns of FLT3-ITD primary AML cells with and without expression of the indicated shRNAs compared to a non-targeting control. G: Pearson correlation between replicates of shRNA knockdown RNA-seq.

Figure S5

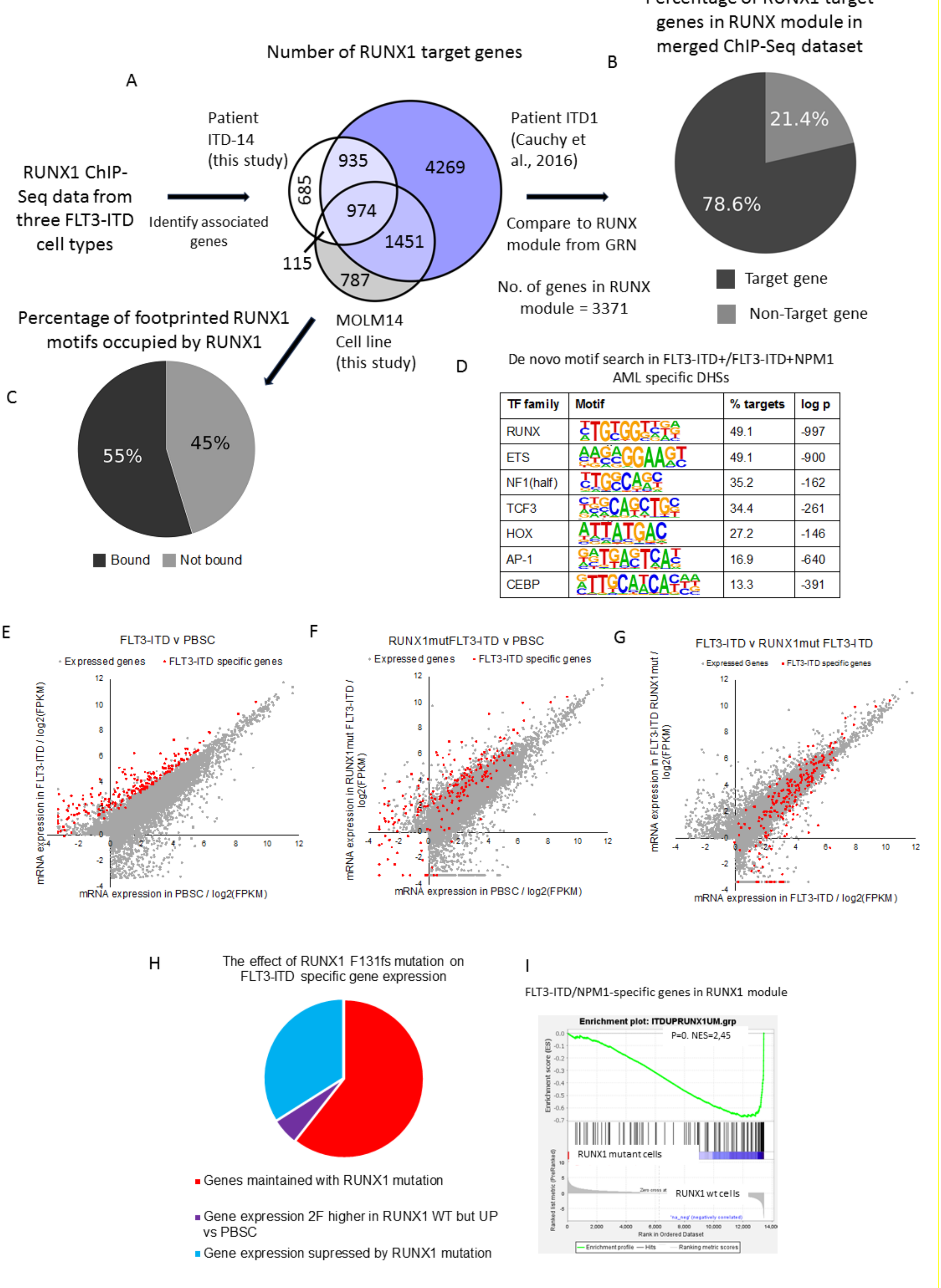

Figure S5 (related to Figure 5) RUNX1 plays a key role in the maintenance of the FLT3-ITD AML phenotype:

A: Venn diagrams showing integration of three RUNX1 ChIP data-sets from primary FLT3-ITD AML cells and cell lines. B: Pie chart showing the percentage of RUNX1 target genes in RUNX module in merged RUNX1 ChIP-Seq dataset. C: Pie chart showing the overlap of footprinted motifs bound by RUNX1 in the ChIP assay.

D: Table of enriched TF motifs in FLT3-ITD AML specific open chromatin regions compared to healthy PBSCs[1]. E: Scatter plot comparing log2(FPKM) gene expression in FLT3-ITD AML to PBSCs. Genes specifically upregulated in ITD+ AML (>2 fold change, p<0.1) are highlighted in red. F: Scatter plot comparing log2(FPKM) gene expression in FLT3-ITD AML with a F131fs RUNX1 mutation[1] to PBSCs. Genes specifically upregulated in ITD+ AML (>2 fold change, p<0.1) are highlighted in red. G: Scatter plot comparing log2(FPKM) gene expression in FLT3-ITD AML to FLT3-ITD AML with F131fs RUNX1 mutation. Genes specifically upregulated in ITD+ AML (>2 fold change, p<0.1) are highlighted in red. H: The effect of a RUNX1 mutation on FLT3-ITD specific gene expression in AML samples. I: GSEA showing the distribution of expression of FLT3-ITD specific genes in the RUNX1 module between AML samples with FLT3-ITD and RUNX1 WT or FLT3-ITD RUNX1 mutant cells.

Figure S6

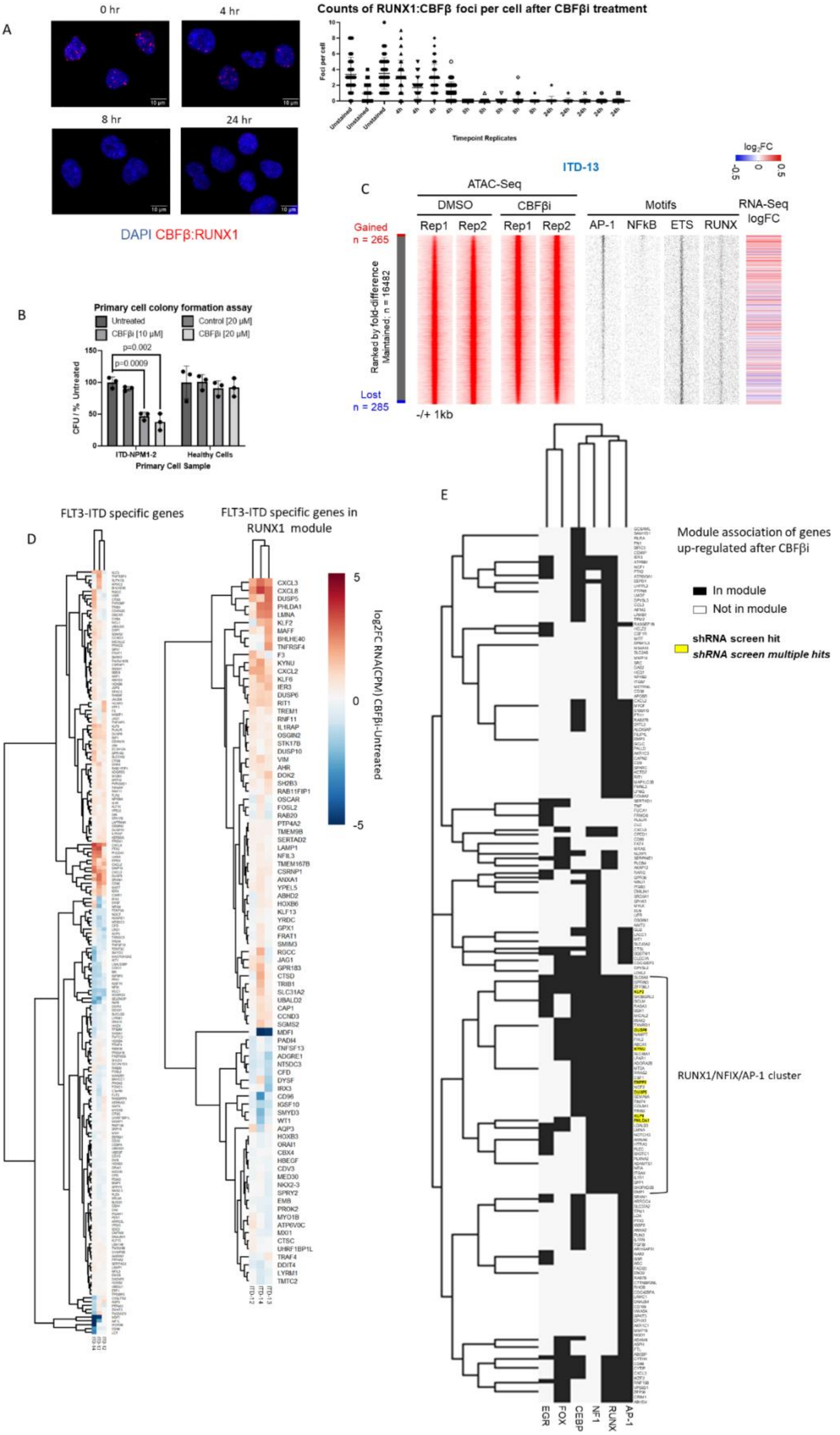
A
0 hr
4 hr
8 hr
24 hr
10 µm
DAPI CBFβ:RUNX1
Counts of RUNX1:CBFβ foci per cell after CBFβi treatment
Foci per cell
Timepoint Replicates
B
Primary cell colony formation assay
Untreated
Control [20 µM]
CBFβi [10 µM]
CBFβi [20 µM]
p=0.002
p=0.0009
CFU / % Untreated
ITD-NPM1-2
Healthy Cells
Primary Cell Sample
C
ITD-13
log2FC
-0.5
0
0.5
ATAC-Seq
DMSO
CBFβi
Rep1
Rep2
Rep1
Rep2
Motifs
AP-1
NFkB
ETS
RUNX
RNA-Seq logFC
Gained n = 265
Ranked by fold-difference Maintained: n = 16482
Lost n = 285
-/+ 1kb
D
FLT3-ITD specific genes
FLT3-ITD specific genes in RUNX1 module
log2FC RNA(CPM) CBFβi-Untreated
5
0
-5
CXCL3
CXCL8
DUSP5
PHLDA1
LMNA
KLF2
MAFF
BHLHE40
TNFRSF4
F3
KYNU
CXCL2
KLF6
IER3
DUSP6
RIT1
TREM1
RNF11
IL1RAP
OSGIN2
STK17B
DUSP10
VIM
AHR
DOK2
SH2B3
RAB11FIP1
OSCAR
FOSL2
RAB20
PTP4A2
TMEM9B
SERTAD2
LAMP1
NFIL3
TMEM167B
CSRNP1
ANXA1
YPEL5
ABHD2
HOXB6
KLF13
YRDC
GPX1
FRAT1
SMIM3
RGCC
JAG1
GPR183
CTSD
TRIB1
SLC31A2
UBALD2
CAP1
CCND3
SGMS2
MDFI
PADI4
TNFSF13
ADGRE1
NT5DC3
CFD
DYSF
IRX3
CD96
IGSF10
SMYD3
WT1
AQP3
HOXB3
ORAI1
CBX4
HBEGF
CDV3
MED30
NKX2-3
SPRY2
EMB
PROK2
MYO1B
ATP6V0C
MXI1
CTSC
UHRF1BP1L
TRAF4
DDIT4
LYRM1
TMTC2
ITD-12
ITD-14
ITD-13
E
Module association of genes up-regulated after CBFβi
In module
Not in module
shRNA screen hit
shRNA screen multiple hits
RUNX1/NFIX/AP-1 cluster
EGR
FOX
CEBP
NF1
RUNX
AP-1

Figure S6 (Related to Figure 6): Perturbation of RUNX1 with CBFβi in FLT3-ITD primary cells
A: Representative images from proximity ligation assay (PLA) showing a time course of dissociation of the CBFβ::RUNX1 complex in primary FLT3-ITD (ITD-14) AML cells after treatment with 10 µM CBFβi. The red signal shows interactions between CBFβ and RUNX1 counterstained with DAPI (blue). Images show these interactions at 4 different time points. The scatter plot shows the number of red foci counted per cell in triplicate at the four different time points. Scale bar = 10 µm. B: Colony forming ability of primary FLT3-ITD AML and healthy cells after treatment with CBFβi. Significant p-values are indicated on the graph and were calculated using Student's t-test. C: Density plot of ATAC-Seq analysis (red) of a second primary FLT3-ITD patient cells (ITD-13) with and without CBFβi ranked against each other according to fold-change with the indicated TF motifs (black) at the open chromatin sites and the logFC RNA expression of the genes associated to the peaks present plotted alongside.
D: Unsupervised clustering of the fold change of gene expression in 3 different patients (ITD-12, ITD-13, ITD-14) after 24 h treatment with 10 µM CBFβi or 0.1% DMSO control. Left panel: FLT3-ITD AML specific genes. Right panel: FLT3-ITD AML specific genes in RUNX1 module.
E: Genes up-regulated in the RNA-seq data in 2 or more of the CBFbi treated patients. Heatmap shows the gene modules associated with each gene (black = associated, white = not). Genes which were single hits in the screen in 1 or more samples are in bold, if there were multiple hits in 1 or more samples they are in italics. Genes not in the RUNX1, AP1, CEBP, EGR1, FOX, NF1 module are not included in this data. Hierarchical clustering was performed to group genes in similar modules.

Figure S7

A

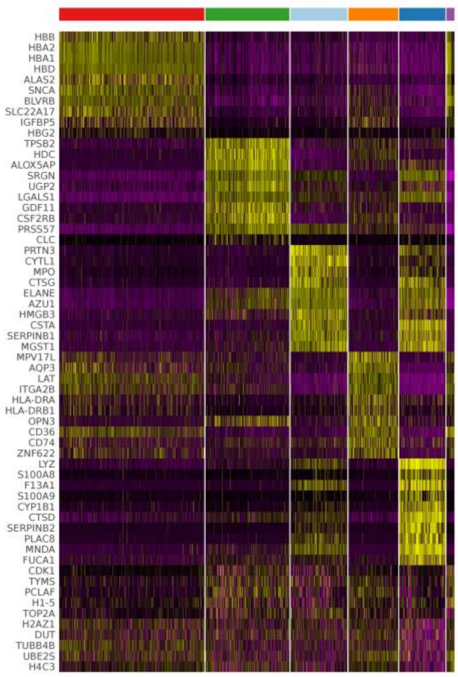

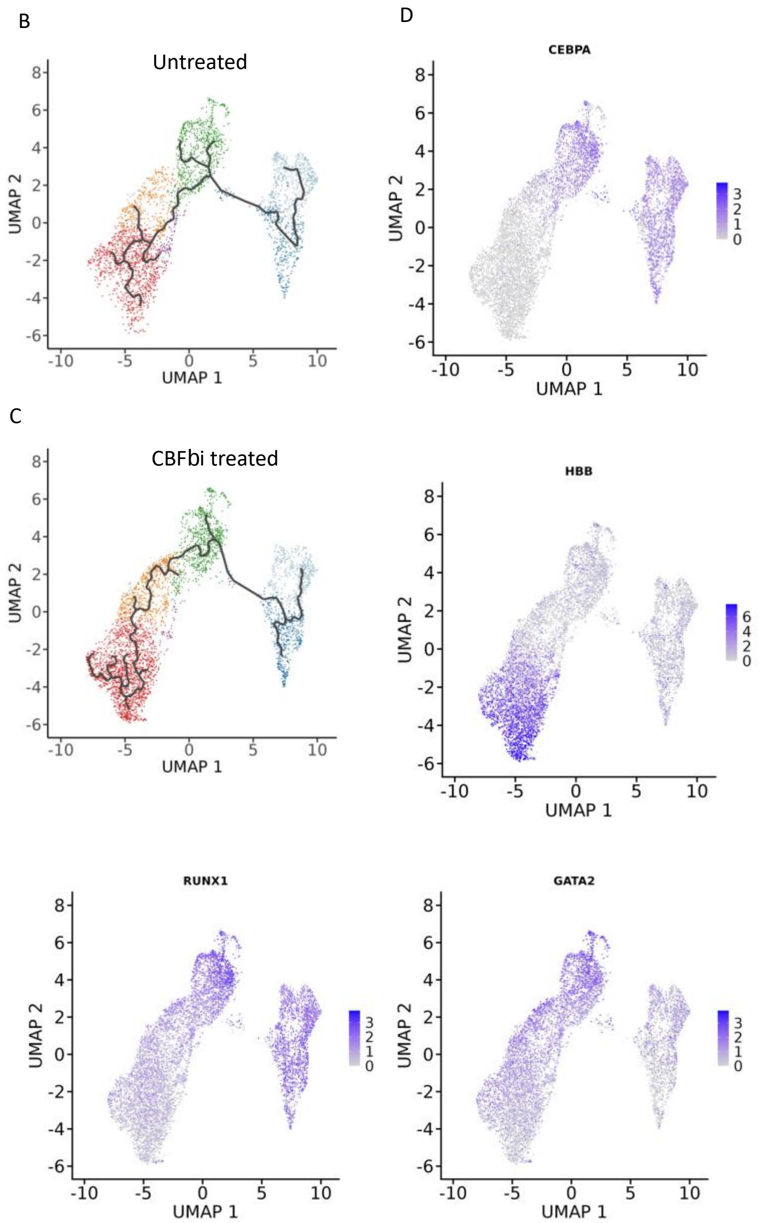

No. of differentially expressed genes per cluster

Cluster ID	Up-Reg	Down-Reg
Early Progenitor	139	271
Eryth Prog - Quiescent	80	121
Eryth Prog - Cycling	27	2
Erythroid	112	131
Early Myeloid	108	246
Myeloid	53	83

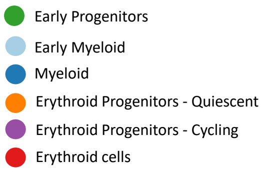

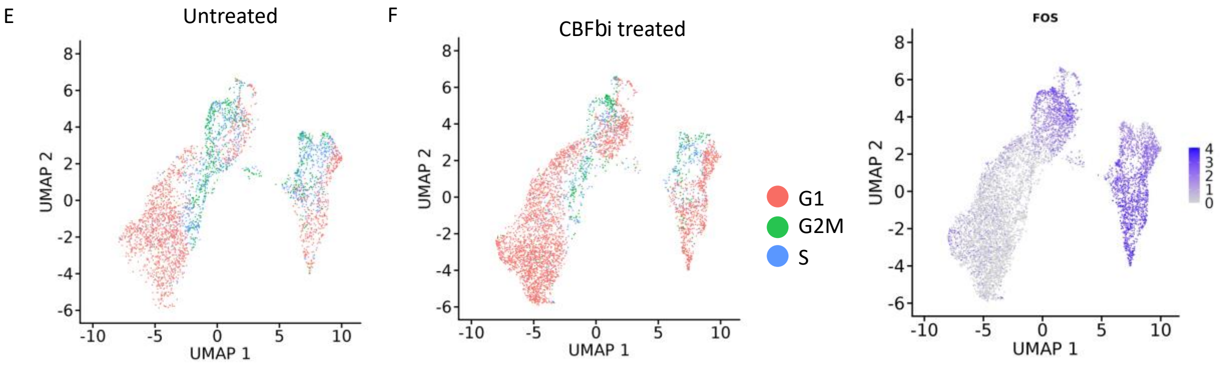

Figure S7 (related to Figure 7): scRNA-Seq analysis of CBFβi treated FLT3-ITD AML patient cells

A: Clustering analysis of scRNA data of the top 10 differentiallly expressed genes in each cluster defining the different cell types in the primary FLT3-ITD AML (ITD-12) culture. B, C: Pseudotime analysis of scRNA data without (B) and with (C) 10 µM CBFβi treatment. D: Expression of the indicated genes in the different cell types plotted onto the UMAP plots. E,F: UMAP plot depicting cell type in the indicated cell cycle phase without (E) and with (F) 10 µM CBFβi treatment.

Supplementary Reference

1. Assi, S.A., Imperato, M.R., Coleman, D.J.L., Pickin, A., Potluri, S., Ptasinska, A., Chin, P.S., Blair, H., Cauchy, P., James, S.R., et al. (2019). Subtype-specific regulatory network rewiring in acute myeloid leukemia. Nat Genet *51*, 151-162. 10.1038/s41588-018-0270-1.
